# Heterostructure of Fe_3_O_4_ Confined in Hierarchical Porous Carbon for Interface‐Enhanced Medical‐Grade H_2_O_2_ Electrosynthesis

**DOI:** 10.1002/advs.202502388

**Published:** 2025-05-19

**Authors:** Yanan Shi, Li‐Li Zhang, Chongyang Wang, Shaohui Sun

**Affiliations:** ^1^ Engineering Research Center of Advanced Functional Material Manufacturing of Ministry of Education School of Chemical Engineering Zhengzhou University Zhengzhou 450001 China; ^2^ State Key Laboratory of Coking Coal Resources Green Exploitation Zhengzhou University Zhengzhou 450001 China; ^3^ Interdisciplinary Research Center for Sustainable Energy Science and Engineering (IRC4SE^2^) School of Chemical Engineering Zhengzhou University Zhengzhou 450001 China

**Keywords:** amorphous carbon, Fe_3_O_4_, heterostructure, hierarchical porous, hydrogen peroxide, oxygen reduction reaction

## Abstract

The electrocatalytic two‐electron oxygen reduction reaction (2e^−^ ORR) presents an environmentally sustainable approach to produce hydrogen peroxide (H_2_O_2_). Heterostructures coupling non‐noble transition metal oxides (TMOs) with carbon materials hold promise for 2e^−^ ORR, but face challenges in controlling morphology, phase composition, and active centers. In this study, a hierarchically porous tremella‐like heterojunction characterized by ultrafine cubic Fe_3_O_4_ nanoparticles within the amorphous carbon (UFe_3_O_4_@HPAC) is obtained using the integrated platform of green Fe‐based deep eutectic solvent via a two‐step annealing process. UFe_3_O_4_@HPAC exhibits remarkable overall and intrinsic 2e^−^ ORR activity, delivering 96% H_2_O_2_ selectivity and a turnover frequency (TOF) of 67.5 s^−1^. Notably, UFe_3_O_4_@HPAC possesses superior H_2_O_2_ production capabilities, showing long‐term stability of 100 h with a H_2_O_2_ production rate of 8.1 g L^−1^ h^−1^ in flow‐cell, while achieving various medical‐grade H_2_O_2_ concentrations (3.0–7.8 wt%). Additionally, integrating on‐site H_2_O_2_ production with electro‐Fenton achieved rapid decomposition of contaminants. The unique heterostructure, with the synergistic effect of Fe_3_O_4_ and amorphous carbon, enhances electronic conductivity. Moreover, the electronic redistribution at the interface of the heterostructure triggers the thermodynamically favorable multiple active sites of Fe and C centers for the 2e^−^ ORR. This work offers a new perspective on transition metal‐based materials for H_2_O_2_ production.

## Graphical abstract

The heterostructure, featuring the interfacial effect of Fe_3_O_4_ and amorphous carbon, enhances electronic conductivity, and activates thermodynamically favorable Fe and C multiple sites for the 2e^–^ ORR by moderately tuning *OOH adsorption strength, enabling efficient medical‐grade H_2_O_2_ synthesis.

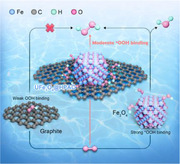

## 1 Introduction

The two‐electron oxygen reduction reaction (2e^−^ ORR) provides a sustainable pathway for hydrogen peroxide (H_2_O_2_) production, driven by renewable energy, with the potential to replace the traditional anthraquinone oxidation method.^[^ ^1^ ^]^ Given that O_2_ can be fully converted into H_2_O via a series of four‐electron steps, developing 2e^−^ ORR catalysts that achieve high activity, selectivity, and stability is a prerequisite for efficient H_2_O_2_ electrosynthesis.^[^ ^2^ ^]^ From a molecular‐level perspective, catalyst materials that exhibit moderate binding energy for *OOH tend to preserve the O─O bond, thereby favoring selectivity toward H_2_O_2_ generation.^[^ ^3^ ^]^ Within this context, non‐noble transition metal‐based materials, including oxides, chalcogenides, and phosphides, have been investigated as 2e^−^ ORR electrocatalysts due to their earth‐abundant nature, environmental compatibility, and tunable composition and structure.^[^ ^4^ ^]^ Among these, transition metal oxides (TMOs) have shown significant 2e^−^ ORR activity, dependent on the inherent crystallographic phase and atomic arrangement.^[^ ^5^ ^]^ By constructing different polymorphic structures of post‐TMOs (ZnO, CuO, NiO, CoO), studies reveal that ZnO, with a larger atomic number, exhibits a moderate adsorption strength for *OOH, approaching the ideal 2e^−^ ORR overpotential.^[^ ^5c^ ^]^ Furthermore, the inert α‐Fe_2_O_3_ was employed to construct specific crystal facets, which optimized the H_2_O_2_ selectivity, while oxygen vacancy engineering was used to modulate the O─O bond dissociation energy, triggering high‐activity sites.^[^ ^6^ ^]^ Similarly, TiO_x_ demonstrated enhanced 2e^−^ ORR activity by altering atomic ordering via oxygen vacancy engineering.^[^ ^7^ ^]^ Despite certain progress, TMOs exhibit low electrical conductivity and specific surface area, which impair electron transfer and limit the exposure of active sites, respectively.

Earth‐abundant carbon‐based materials have superior intrinsic conductivity and diverse porous structures, benefiting from enhancing electron transfer ability and facilitating mass and ion transport.^[^ ^1b^ ^]^ Therefore, the construction of heterostructures coupling TMOs with carbon‐based materials tends to present distinct physicochemical, geometric, and crystallographic properties. This strategy has emerged as an effective means to enhance the performance of TMOs in 2e^−^ ORR electrocatalysis.^[^ ^4^, ^8^ ^]^ In view of this, Wu and colleagues prepared amorphous nickel oxide (NiO_x_) supported on carbon nanosheets, which delivered a high H_2_O_2_ selectivity of 91% and a low onset potential of 0.76 V vs RHE.^[^ ^8^ ^]^ Cao et al. and Wang et al. have demonstrated that the embedding iron‐based oxides (e.g., FeO, Fe_2_O_3_, or FeO_x_) into carbon matrices in a heterostructure effectively facilitates the 2e^−^ mediated ORR.^[^ ^9^ ^]^ Similarly, the Fe_3_O_4_‐dominated crystalline nitrogen‐doped carbon catalyst, anchoring Fe_3_O_4_ and metallic Fe, achieves thermodynamically optimal *OOH binding and an *OOH‐mediated fast reaction pathway.^[^ ^10^ ^]^ Nevertheless, most TMOs heterostructures directly supported on carbon substrates exhibit relatively weak long‐term mechanical durability. Additionally, the morphology and phase composition of TMOs are challenging to control, leading to aggregation and phase complexity, in turn obscuring the precise identification of active sites and their roles in catalytic performance. Therefore, developing TMOs heterojunctions with strong adhesion, optimized composition, and morphology to promote 2e^−^ ORR performance and investigate their impact remains a significant challenge.

Herein, a heterojunction characterized by ultrafine cubic Fe_3_O_4_ nanoparticles within the amorphous carbon (UFe_3_O_4_@HPAC) was constructed using the integrated platform of green Fe‐based deep eutectic solvent via a two‐step annealing process. The UFe_3_O_4_@HPAC heterojunction exhibits remarkable overall and intrinsic activity of the 2e^−^ ORR, delivering a high H_2_O_2_ selectivity of 96% along with an impressive turnover frequency (TOF) of 67.5 s^−1^ at 0.6 V vs RHE. Furthermore, the UFe_3_O_4_@HPAC electrode achieved the long‐term operation of 100 h with an excellent H_2_O_2_ production rate of 8.1 g L^−1^ h^−1^ in flow‐cell, while achieving H_2_O_2_ concentrations suitable for various medical applications (3.0–7.8 wt%). Moreover, integrating the electrocatalytic 2e^−^ ORR with the electro‐Fenton process achieved high degradation efficiency of rhodamine B (RhB) and methylene blue (MB) contaminants. Theoretical studies and electrochemical analyses reveal that the unique heterostructure with Fe_3_O_4_ and the carbon matrix optimizes the electronic structure, enhancing electronic conductivity, and stabilizing the *OOH intermediate, thereby triggering thermodynamically advantageous multiple active sites for the 2e^−^ ORR.

## 2 Results and Discussion

### 2.1 Probing Physicochemical Structure of Catalysts

The material was synthesized using a two‐step annealing approach via a green multifunctional platform of Fe‐based deep eutectic solvent (PEG200/FeCl_3_·6H_2_O‐DES) containing macromolecular polyethylene glycol (PEG200) (Figure S1a, Supporting Information). To stabilize the metal species and prevent aggregation in subsequent steps, the first‐step annealing temperature (*T_1_*) should be below the complete decomposition temperature of PEG200/FeCl_3_·6H_2_O‐DES for promoting partial transformation (Figure S1b, Supporting Information). Intermediate products, obtained after washing to remove unconverted species and unfixed iron species in the first annealing step, were monitored for their phase transitions as the temperature increased using in situ X‐ray diffraction (XRD). As displayed in Figure S1c (Supporting Information), no crystalline phases were detected in the intermediate products. However, upon heating to 400 °C, diffraction peaks indicative of Fe_3_O_4_ appeared, while no additional iron species were formed or transformed with further temperature increases and extended annealing time. Therefore, in the second annealing step, a higher temperature is required to complete carbonization and crystallization to obtain the target material UFe_3_O_4_@HPAC. For comparison, the carbon material HPAC was derived from UFe_3_O_4_@HPAC following hydrogen reduction and acid leaching, with its iron species removal validated by inductively coupled plasma mass spectrometry (ICP‐MS) and energy dispersive X‐ray (EDX) analysis (Figure S2 and Table S1, Supporting Information). Single‐phase Fe_3_O_4_ was prepared from FeCl_3_·6H_2_O via the one‐step annealing processes, as confirmed by the XRD pattern (Figure S3, Supporting Information). The XRD pattern of UFe_3_O_4_@HPAC reveals the presence of amorphous carbon and Fe_3_O_4_ phases, with no additional phases detected (Figure S3, Supporting Information). Additionally, HPAC exists in the form of amorphous carbon, characterized by two typical wide peaks of the (002) and (101) planes at 26° and 43°.^[^ ^11^ ^]^

**Figure 1:**
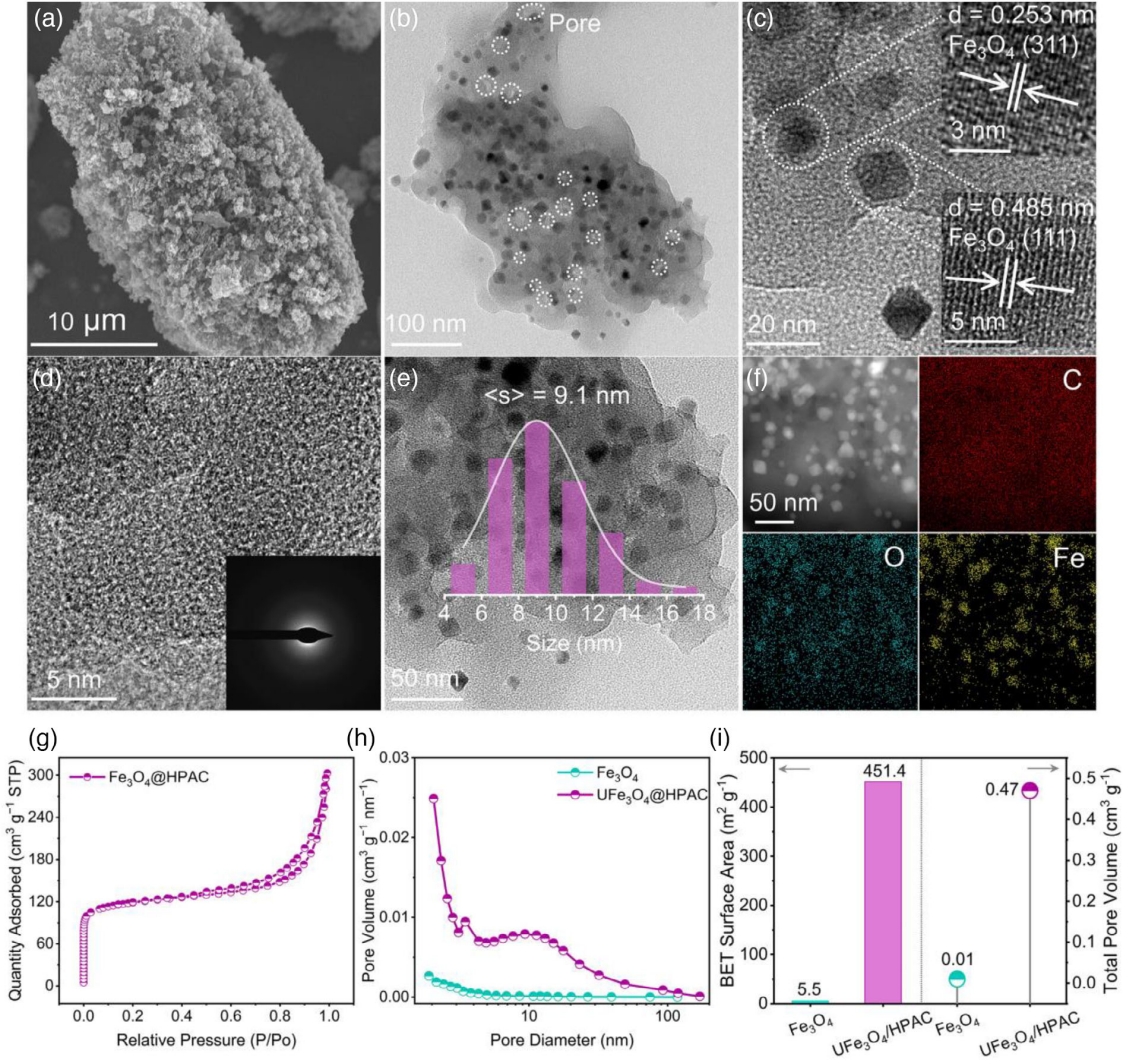
a) SEM, b) TEM, and c,d) HRTEM images of UFe_3_O_4_@HPAC. e) Particle size distribution in UFe_3_O_4_@HPAC. f) HAADF‐STEM image along with corresponding elemental mapping of UFe_3_O_4_@HPAC. g) N_2_ adsorption–desorption isotherms of UFe_3_O_4_@HPAC. h) Pore size distribution, i) BET surface area, and total pore volume for the UFe_3_O_4_@HPAC and Fe_3_O_4_ catalysts.

The surface structures of catalysts were first studied by scanning electron microscopy (SEM) and transmission electron microscopy (TEM). SEM images revealed that the tremella‐like morphology of UFe_3_O_4_@HPAC is constructed from rough‐surface nanoplates (**Figures** **1**a and S4, Supporting Information), in contrast to the smooth‐surface irregular block‐like structure of the Fe_3_O_4_ catalyst (Figures S5a and S6, Supporting Information). Furthermore, a hierarchical porous microstructure, ranging from 3 to 20 nm, was observed in the TEM image of UFe_3_O_4_@HPAC (white dashed circle, Figure 1b), which is expected to enhance the surface area of the material. Nevertheless, the HPAC that was obtained from UFe_3_O_4_@HPAC through hydrogen reduction and acid treatment had a collapsed nanosheet structure (Figure S5b, Supporting Information). The microstructures of the catalysts were further analyzed using high‐resolution TEM (HRTEM). Figure 1c reveals the lattice spacings of 0.253 and 0.485 nm in UFe_3_O_4_@HPAC, corresponding to the (311) and (111) planes of Fe_3_O_4_, respectively.^[^ ^12^ ^]^ Moreover, the carbon matrix exists as amorphous carbon, consistent with the XRD analysis (Figure 1d). Notably, a heterojunction, characterized by ultrafine cubic Fe_3_O_4_ nanoparticles (≈9.1 nm) uniformly distributed within the amorphous carbon matrix, was observed (Figure 1e and Figure S7, Supporting Information), likely caused by the effective inhibition of Fe_3_O_4_ aggregation by PEG200 during the two‐step annealing process. These observations were further corroborated by aberration‐corrected high‐angle annular dark‐field scanning TEM (HAADF‐STEM) images and EDX mappings (Figure 1f). The distribution of Fe atoms in UFe_3_O_4_@HPAC is highly consistent with the Fe_3_O_4_ nanoparticles. The O element is concentrated in the Fe_3_O_4_ nanoparticle regions, with a more uniform distribution in other areas, while the C element exhibits a uniformly distributed pattern across the material. The unique structure is anticipated to mitigate metal leaching during catalytic reactions, thereby facilitating long‐term stability.

To further analyze the structural characteristics of the catalysts, including pore architecture and surface properties, N_2_ adsorption–desorption measurements were performed. The isotherms of UFe_3_O_4_@HPAC display both type I and IV hysteresis loops, indicating the formation of a unique hierarchical porous structure (Figure 1g).^[^ ^13^ ^]^ Compared to the virtually non‐porous distribution of the Fe_3_O_4_ catalyst, UFe_3_O_4_@HPAC exhibits a wide pore distribution ranging from 0.6 to 92.5 nm (Figure 1h and Figure S9d, Supporting Information). Moreover, the surface area of UFe_3_O_4_@HPAC is 451.4 m^2^ g^−1^, accompanied by a total pore volume of 0.47 cm^3^ g^−1^, both of which are significantly higher than those of the Fe_3_O_4_ catalyst, being 82 and 47 times higher, respectively (Figure 1i). This particular morphology of UFe_3_O_4_@HPAC may provide additional active sites and diffusion channels, thereby facilitating the rapid O_2_ conversion and H_2_O_2_ transfer in 2e^−^ ORR, preventing local aggregation and side reactions, and ultimately improving catalytic activity.^[^ ^13b^ ^]^

**Figure 2:**
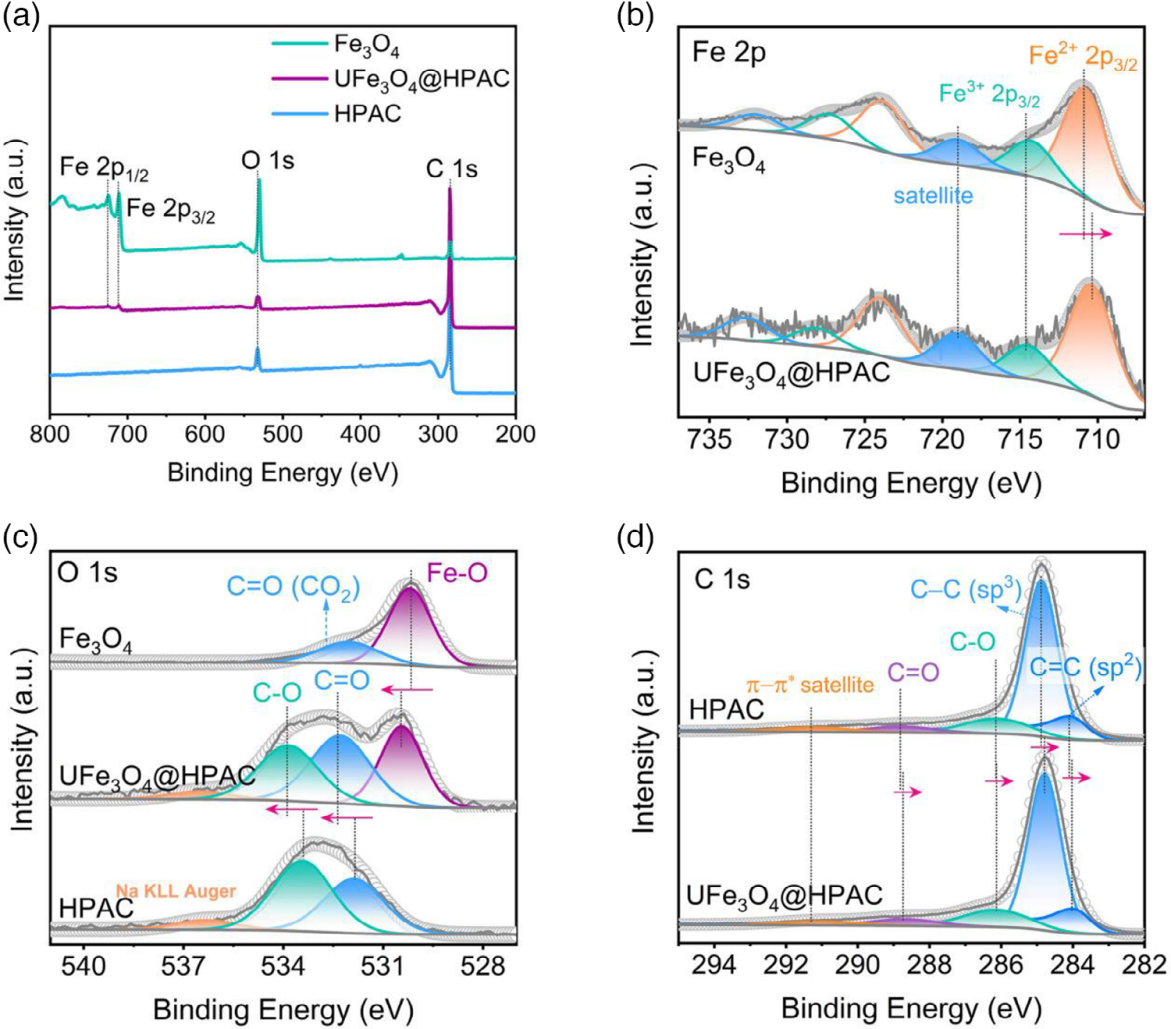
a) XPS survey spectra of HPAC, UFe_3_O_4_@HPAC, and Fe_3_O_4_. b) High‐resolution Fe 2p XPS spectra of UFe_3_O_4_@HPAC and Fe_3_O_4_. c) High‐resolution O 1s XPS spectra of HPAC, UFe_3_O_4_@HPAC, and Fe_3_O_4_. d) High‐resolution C 1s XPS spectra of HPAC and UFe_3_O_4_@HPAC.

Raman spectroscopy and X‐ray photoelectron spectroscopy (XPS) were employed to investigate possible structural modifications and chemical states within the catalysts. According to Raman spectroscopy, the I_D_/I_G_ intensity ratio of UFe_3_O_4_@HPAC (0.70) is similar to that of the HPAC catalyst (0.69),^[^ ^14^ ^]^ which suggests that defects may not significantly influence catalyst performance (Figure S10, Supporting Information). The XPS survey verifies the absence of Fe on the HPAC surface, contrasting with the Fe element content of 0.78 at% in UFe_3_O_4_@HPAC (**Figure** **2**a and Table S2, Supporting Information). Figure 2b depicts the core‐level XPS at the Fe 2p region, where the binding energy for Fe^2+^ 2p_3/2_ negatively shifts from 710.79 eV on Fe_3_O_4_ to 710.36 eV UFe_3_O_4_@HPAC,^[^ ^15^ ^]^ implying more electron depletion from the neighboring O or C sites to the Fe atomic centers of UFe_3_O_4_@HPAC. Correspondingly, deconvolution of the O 1s spectra revealed the Fe–O peak for UFe_3_O_4_@HPAC presented a higher binding energy than that of the Fe_3_O_4_ catalyst (Figure 2c).^[^ ^15b^ ^]^ Noteworthy, the C─O and C═O peaks of O 1s in UFe_3_O_4_@HPAC also red‐shifted to a higher binding energy compared to that of the HPAC catalyst (Figure 2c), meanwhile no Fe–C peak is observed in C 1s of UFe_3_O_4_@HPAC (Figure 2d),^[^ ^16^ ^]^ indicating metal coordination interactions between Fe atoms and O atoms on the carbon matrix, rather than with carbon atoms. Furthermore, C = C (sp^2^), C─C (sp^3^), CɐO, and C═O in the C 1s XPS spectra of UFe_3_O_4_@HPAC shift toward lower binding energy relative to HPAC, indicating electron enrichment in the carbon matrix and a consequent reduction in its electronegativity.^[^ ^17^ ^]^ This enhances its ability to donate electrons to reactants, promoting their adsorption, while possibly optimizing the reaction pathway to facilitate product desorption and accelerate reaction kinetics.

Building upon the above analyses and TEM results, the heterojunction UFe_3_O_4_@HPAC exhibits interfacial effects between Fe_3_O_4_ nanoparticles and the amorphous carbon matrix. The interaction may enhance the mechanical strength of Fe_3_O_4_ within the carbon matrix, thereby improving the long‐term stability of the material during three‐phase interfacial reactions. Moreover, the electronic rearrangement of Fe, O, and C for UFe_3_O_4_@HPAC likely activates the high‐activity sites, facilitating the efficient conversion of O_2_ to H_2_O_2_.

### 2.2 Intrinsic 2e^−^ ORR Performance Evaluation

**Figure 3:**
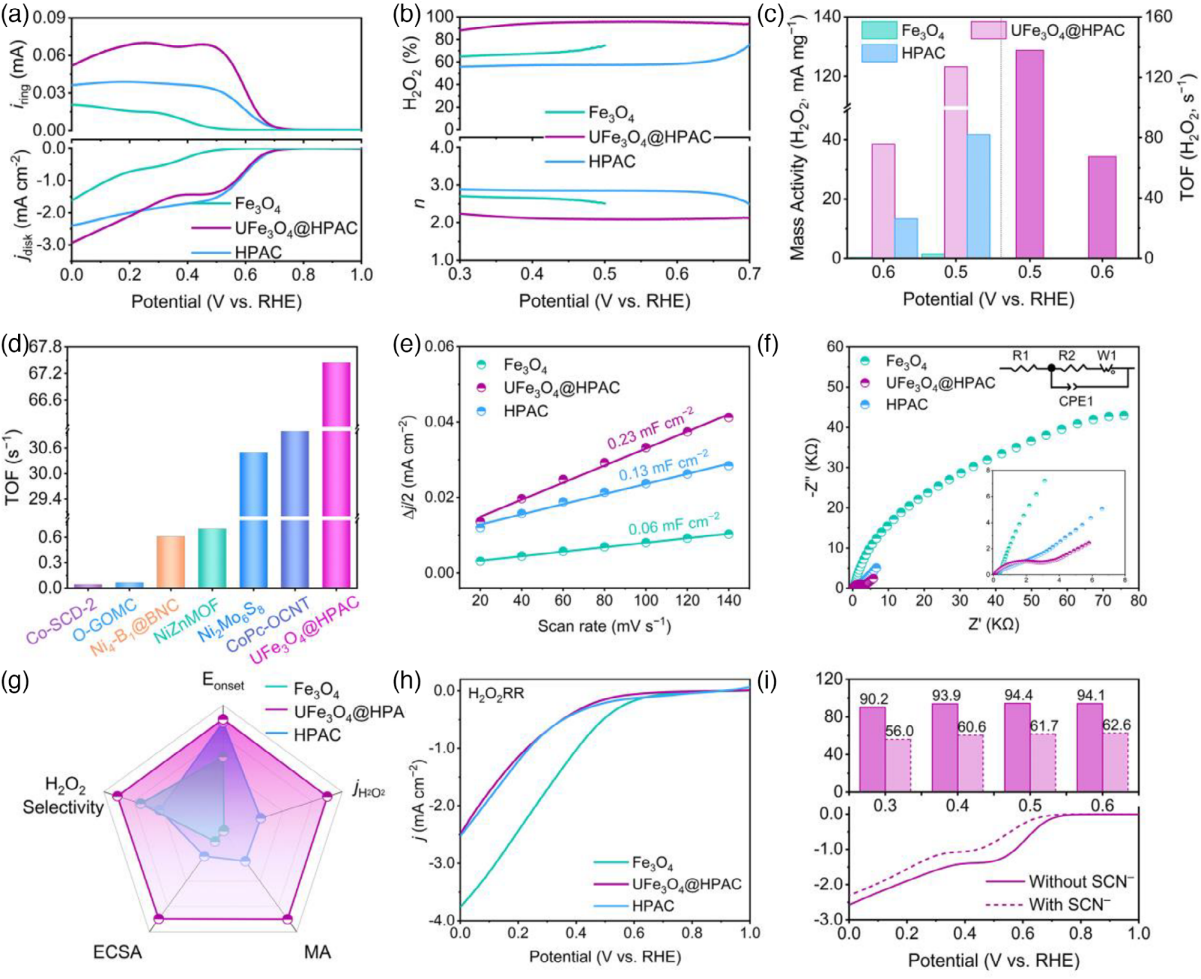
a) LSV curves of the synthesized catalysts at 1600 rpm, recorded at a scan rate of 5 mV s^−1^ in 0.1 m KOH. b) H_2_O_2_ selectivity and *n* of the synthesized carbon catalysts, derived from the LSV data. c) Mass activity of H_2_O_2_ production for HPAC, UFe_3_O_4_@HPAC, and Fe_3_O_4_, and TOF of UFe_3_O_4_@HPAC. d) Comparison of TOF for the UFe_3_O_4_@HPAC and previously reported catalysts. e) *C*_dl_ of the prepared catalysts based on CV curves. f) Nyquist plots. g) Radar chart depicting the ORR performance of the prepared catalysts. h) LSV curves of the H_2_O_2_RR. i) ORR LSV curves and H_2_O_2_ selectivity for UFe_3_O_4_@HPAC before and after the addition of SCN^−^ ions to the solution in an O_2_‐saturated 0.1 m KOH.

The 2e^−^ ORR performance of the catalysts with the different chemistries and electronic structures was assessed in 0.1 m KOH using a rotating ring–disk electrode (RRDE) setup. The cyclic voltammograms of Fe_3_O_4_, UFe_3_O_4_@HPAC, and HPAC in O_2_‐saturated 0.1 m KOH display distinct peaks for oxygen reduction, while no peaks are observed under N_2_ saturation (Figure S11, Supporting Information).^[^ ^5c^ ^]^ Among them, UFe_3_O_4_@HPAC exhibits the highest current density at a more positive peak potential of 0.57 V vs RHE, indicating its superior catalytic activity for oxygen reduction. Upon comparing the linear sweep voltammograms (LSVs) recorded for all catalysts (**Figure** **3**a), the UFe_3_O_4_@HPAC heterojunction, composed of Fe_3_O_4_ and amorphous carbon, delivers a significantly earlier onset potential (0.71 V vs RHE) than that of the Fe_3_O_4_ catalyst (0.47 V vs RHE), implying enhanced electrical conductivity with the introduction of amorphous carbon. Likewise, the disk current density (*j*_disk_) of UFe_3_O_4_@HPAC has been greatly improved with a similar *j*_disk_ to that of the HPAC catalyst. Notably, UFe_3_O_4_@HPAC exhibits the most prominent ring current (*i*_ring_) among all catalysts; however, a dramatic drop in the *i*_ring_ is observed for the HPAC catalyst without Fe_3_O_4_ species, revealing that Fe_3_O_4_ may regulate the preference of UFe_3_O_4_@HPAC toward the 2e^−^ ORR pathway. Correspondingly, the H_2_O_2_ selectivity of UFe_3_O_4_@HPAC also remains the highest across the entire potential range, reaching a value of 96% (Figure 3b), among the top‐performing reported transition metal‐based catalysts for the 2e^−^ ORR (Table S3, Supporting Information). Additionally, the electron transfer number (*n*) for UFe_3_O_4_@HPAC is ≈2.1 consistent with the results obtained from the K‐L equation calculation (Figure S12, Supporting Information), further indicating that UFe_3_O_4_@HPAC predominantly follows the 2e^−^ pathway of ORR. To better assess the intrinsic activity of catalysts, mass activity (MA_H2O2_) and turnover frequency (TOF) of H_2_O_2_ production were analyzed (Figure 3c). As the applied potential shifts to more negative values, the MA_H2O2_ for all catalysts exhibits an increase. Specifically, the MA_H2O2_ of UFe_3_O_4_@HPAC is 38.5 mA mg^−1^ at 0.6 V vs RHE, surpassing Fe_3_O_4_ and HPAC by factors of 128 and 3, respectively. Furthermore, the TOF of UFe_3_O_4_@HPAC is as high as 67.5 s^−1^ at 0.6 V vs RHE, outperforming previously reported O_2_–to–H_2_O_2_ conversion performance (Figure 3d).^[^ ^18^ ^]^ Impressively, the ORR activity and selectivity of UFe_3_O_4_@HPAC remain stable over 5000 repeated CV cycles during the accelerated degradation test (Figure S13a–c, Supporting Information). More importantly, the heterojunction structure is retained after prolonged operation, as evidenced by the uniform distributed Fe_3_O_4_ nanoparticle within the amorphous carbon matrix (Figure S13d–f, Supporting Information) and the negligible leaching of metallic iron (Table S4, Supporting Information). Collectively, these results highlight that the unique heterojunction structure formed by Fe_3_O_4_ and the amorphous carbon not only enhances the overriding activity of the 2e^−^ ORR but also promotes the intrinsic activity of the active sites, ensuring long‐term stability.

Figure S14 (Supporting Information) shows the Tafel plots derived from LSV data to evaluate the ORR catalytic kinetics, where UFe_3_O_4_@HPAC exhibits the most favorable kinetics with the lowest Tafel slope. Electrochemically active surface area (ECSA) was investigated for the intrinsic activities of these catalysts. Figure 3e illustrates that the UFe_3_O_4_@HPAC heterojunction exhibits a higher ECSA, in line with the BET surface area, suggesting that it provides more active centers than the Fe_3_O_4_ and HPAC catalysts. The H_2_O_2_ current density normalized by ECSA (*j*_H2O2, ECSA_) is another critical metric for evaluating the intrinsic activity of catalysts in the 2e^−^ ORR. Notably, UFe_3_O_4_@HPAC exhibits the highest *j*_H2O2, ECSA_ (Figure S15, Supporting Information), further corroborating that the synergistic interaction between Fe_3_O_4_ and amorphous carbon effectively activates the highly active sites of the 2e^−^ ORR. Moreover, in the electrochemical impedance spectroscopy (EIS) measurements, a smaller semicircle diameter for the UFe_3_O_4_@HPAC heterojunction was observed in the high‐frequency region, in clear contrast to that of the Fe_3_O_4_ catalyst (Figure 3f). This result indicates that the synergistic effect of Fe_3_O_4_ and amorphous carbon promotes the electron transfer capability of the UFe_3_O_4_@HPAC heterojunction, which further corroborates the conclusions drawn from the 2e^−^ ORR performance analysis (Figure 3g).

To better understand the contribution of Fe species to the 2e^−^ ORR, the H_2_O_2_ reduction reaction (H_2_O_2_RR) was conducted on all catalysts (Figure 3h), where UFe_3_O_4_@HPAC and Fe_3_O_4_ displayed lower H_2_O_2_RR activity than that of HPAC without Fe_3_O_4_. Subsequently, a control experiment on Fe site poisoning by SCN^−^ reveals that the significantly and moderately decayed ring and disk current were noticed on UFe_3_O_4_@HPAC,^[^ ^19^ ^]^ leading to a decrease in H_2_O_2_ selectivity (Figure 3i). These results indicate Fe_3_O_4_ nanoparticles in UFe_3_O_4_@HPAC facilitate 2e^−^ pathway of ORR, while effectively inhibiting the excessive reduction of H_2_O_2_.

### 2.3 Electrochemical H_2_O_2_ Synthesis Evaluation

**Figure 4:**
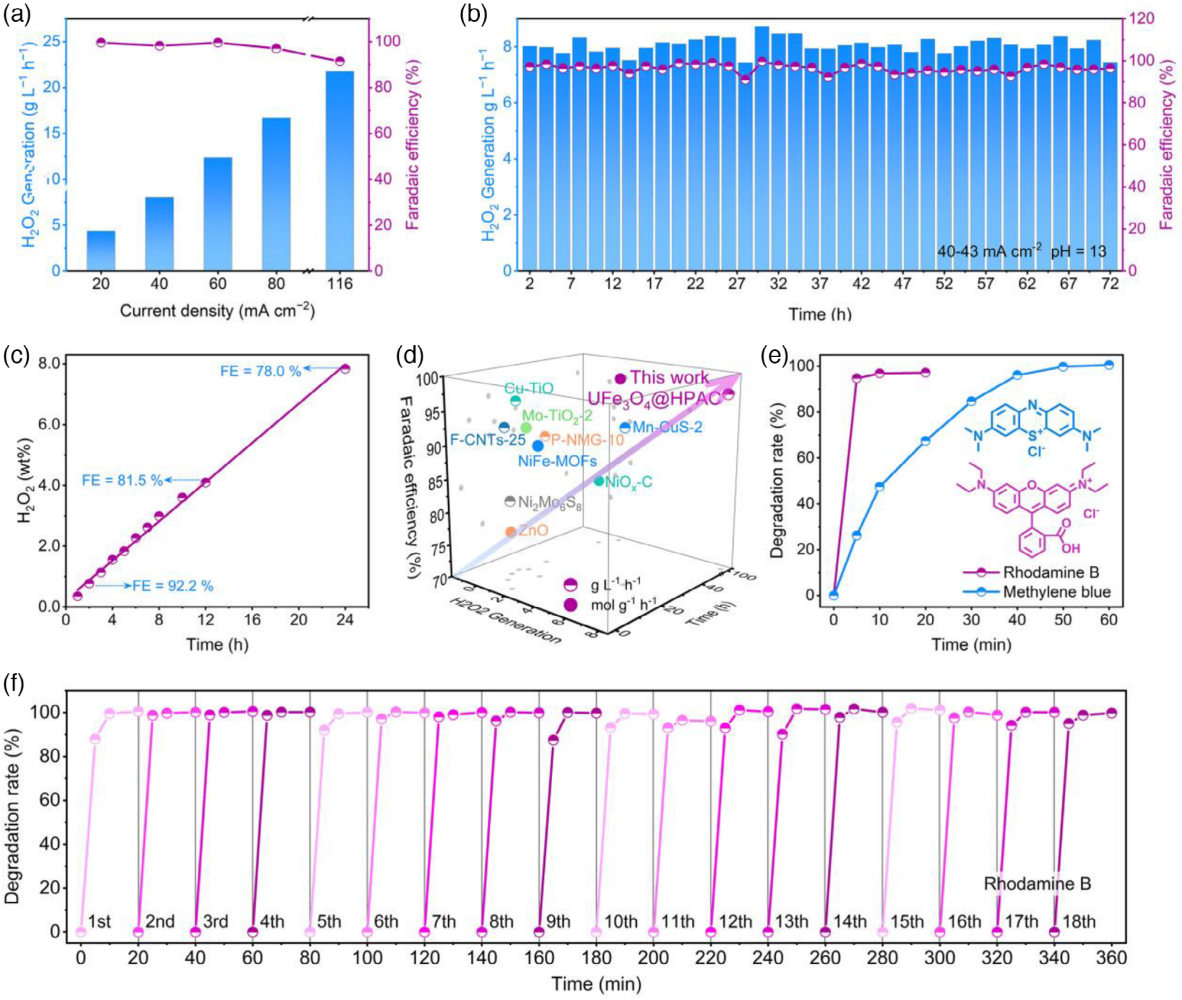
a) FE and H_2_O_2_ production rate of UFe_3_O_4_@HPAC at different current densities. b) FE and H_2_O_2_ production rate of UFe_3_O_4_@HPAC over 72 h in 0.5 m Na_2_SO_4_ and 0.1 m KOH. c) Accumulated H_2_O_2_ concentration of UFe_3_O_4_@HPAC over 24 h in 0.5 m Na_2_SO_4_ and 0.1 m KOH. d) H_2_O_2_ production rate, FE and stability of UFe_3_O_4_@HPAC, along with the reported catalysts in alkaline media. e) Degradation efficiency of 500 mg L^−1^ RhB and MB over time in 0.5 m Na_2_SO_4_ (pH = 3). f) Stability evaluation over 18 consecutive cycles for the degradation of 500 mg L^−1^ RhB.

A two‐electrode flow cell was employed to assess the ability to electrosynthesis H_2_O_2_, with a Pt wire serving as the anode and a proton exchange membrane (PEM, Nafion 117) dividing the anode and cathode compartments. The optimal performance UFe_3_O_4_@HPAC catalyst was mixed with the binder polytetrafluoroethylene (PTFE) to form a slurry, which was then coupled with a gas diffusion electrode (GDE). After annealing, the UFe_3_O_4_@HPAC GDE electrode assembly was used as the working electrode. This process effectively preserved the phase composition of Fe_3_O_4_ and amorphous carbon in UFe_3_O_4_@HPAC (Figure S16a, Supporting Information). Additionally, PTFE enhances the hydrophobicity of the UFe_3_O_4_@HPAC GDE (Figure S16b, Supporting Information), which facilitates efficient contact between O_2_ and catalytically active sites while preventing excessive water accumulation on the catalyst surface, thereby contributing to the long‐term stability of the electrode.^[^ ^19^ ^]^ As displayed in **Figure** **4**a, the UFe_3_O_4_@HPAC GDE achieved a high Faradaic efficiency (FE) of ≈100% over a wide current density range, accompanied by a gradual increase in the H_2_O_2_ production rate. Moreover, the FE still remains at 92% at an industry‐relevant current density of 118 mA cm^−2^.^[^ ^20^ ^]^ Thereafter, this UFe_3_O_4_@HPAC GDE was further continuously operated for 72 h with the electrolyte being refreshed every 2 h, achieving a stable H_2_O_2_ concentration of 1.6 wt%, comparable to the initial concentrations (1–2 wt%) produced industrially via the anthraquinone process (Figure 4b).^[^ ^21^ ^]^ Throughout this period, a remarkable H_2_O_2_ production rate of 8.1 g L^−1^ h^−1^ and a corresponding Faradaic efficiency (FE) exceeding 96% were attained. Notably, continuous operation of the same UFe_3_O_4_@HPAC GDE at low current densities yielded H_2_O_2_ suitable for medical or disinfectant applications, achieving concentrations of 3.0, 4.1, and 7.8 wt% after 8, 12, and 24 h of operation, respectively (Figure 4c).^[^ ^21^ ^]^ Finally, the UFe_3_O_4_@HPAC GDE maintained excellent O_2_‐to‐H_2_O_2_ conversion over 100 h, exhibiting superior H_2_O_2_ production capabilities among the reported catalysts (Figure 4d),^[^ ^1^, ^5^, ^8^, ^13^, ^18^, ^22^ ^]^ highlighting its potential for electrochemical 2e^−^ ORR.

Motivated by the efficient on‐site H_2_O_2_ production by the UFe_3_O_4_@HPAC catalyst, we explored its potential in the electro‐Fenton processes for degrading organic pollutants, with rhodamine B (RhB) and methylene blue (MB) serving as model contaminants. In this process, H_2_O_2_ reacts with Fe^2+^ via the electro‐Fenton process, producing hydroxyl radicals (·OH) that effectively degrade organic contaminants.^[^ ^23^ ^]^ The complete degradation of RhB and MB at concentrations of 500 mg L^−1^ occurred within 20 and 60 min at a constant current of 40 mA cm^−2^ (Figure 4e and Figure S17, Supporting Information), respectively. Under nitrogen‐saturated conditions, 500 mg L^−1^ RhB exhibited negligible degradation within 10 min (Figure S18a,b, Supporting Information); moreover, the adsorption degradation on the UFe_3_O_4_@HPAC GDE remained as low as 3.1% over 30 min (Figure S18c,d, Supporting Information), underscoring the indispensable role of electrocatalytic H_2_O_2_ generation in the electro‐Fenton process. Importantly, the degradation efficiency of 500 mg L^−1^ RhB was consistently maintained at 100% within 20 min over 18 successive cycles (Figure 4f); meanwhile, the heterojunction structure of UFe_3_O_4_@HPAC was well preserved (Figure S19, Supporting Information). These outcomes point to the fact that the UFe_3_O_4_@HPAC catalyst demonstrates the potential for practical applications in wastewater treatment.

### 2.4 Computational Analysis

To gain insight into the atomic‐level synergistic interaction between Fe_3_O_4_ and amorphous carbon, density functional theory (DFT) calculations were carried out. Based on the XRD and HRTEM results, Fe_3_O_4_(111) and Fe_3_O_4_(220) are modeled as representative surfaces of the Fe_3_O_4_ catalyst, while monolayer graphene, referred to as Graphite in the model, represents the HPAC catalyst (Figure S20, Supporting Information). Additionally, UFe_3_O_4_@HPAC consists of a heterojunction formed between monolayer graphene and Fe_3_O_4_(111) or Fe_3_O_4_(220), denoted as Fe_3_O_4_(111)‐gra, and Fe_3_O_4_(220)‐gra, respectively (Figure S21, Supporting Information).

**Figure 5:**
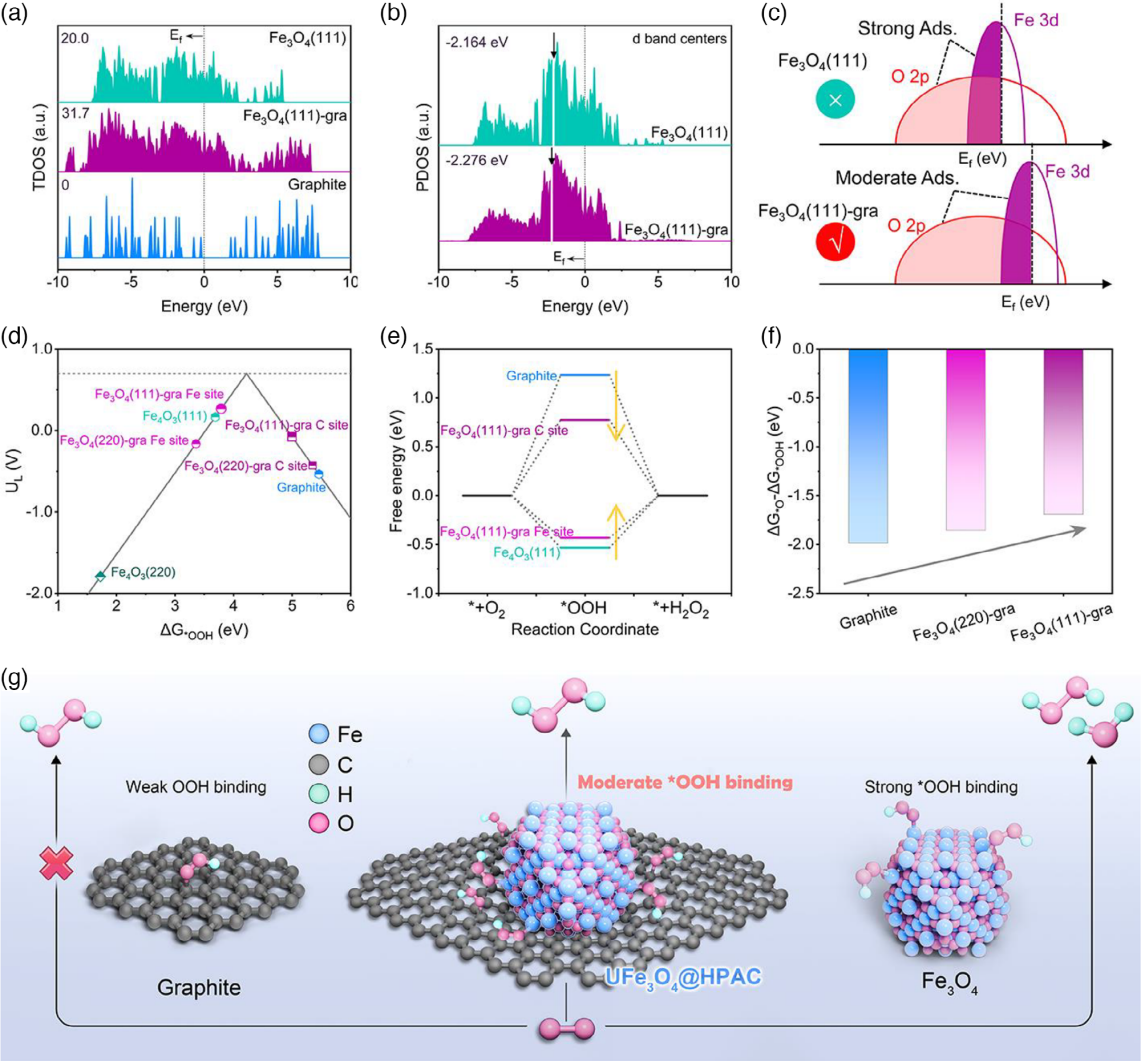
a) TDOS for various models. b) D‐band center for Fe_3_O_4_(111) and Fe_3_O_4_(111)‐gra models. c) Energy band structure diagrams for Fe_3_O_4_(111) and Fe_3_O_4_(111)‐gra. d) Volcano plot showing the relationship between U_L_ and ΔG_*__OOH_. e) Free energy diagrams for distinct models for the 2e^−^ ORR pathways at an equilibrium potential of 0.7 V vs RHE. f) ΔG_*__O_‐ΔG_*__OOH_ value of different models. g) Diagrammatic representation of the 2e^−^ ORR mechanism for different catalysts.

Total Density of States (TDOS) reveals that Fe_3_O_4_(111)‐gra heterojunction demonstrates distinctly enhanced electronic conductivity compared to Fe_3_O_4_(111) and Graphite, as evidenced by the higher electron density around the Fermi level and the broader electronic distribution in Fe_3_O_4_(111)‐gra (**Figure** **5**a).^[^ ^24^ ^]^ Additionally, the electron density of C 2p partial DOS (PDOS) in Fe_3_O_4_(111)‐gra crosses the Fermi energy level, unlike Graphite (Figure S22, Supporting Information), which validates that Fe_3_O_4_ optimizes the electronic structure of the carbon matrix, likely facilitating electron transition from the valence band to the conduction band. This optimization enhances O_2_ adsorption on carbon sites of Fe_3_O_4_(111)‐gra and promotes further protonation, thereby accelerating the 2e^−^ ORR kinetics. Furthermore, a downward shift in the d‐band energy level of Fe_3_O_4_(111)‐gra relative to Fe_3_O_4_(111) is observed (Figure 5b). This shift suggests a greater filling of antibonding orbitals, leading to a higher population of Fe 3d orbitals and consequently weakening the interaction with O 2p orbitals.^[^ ^18b^ ^]^ It is widely recognized that a moderate metal‐oxygen bond strength facilitates favorable oxygen reactivity (Figure 5c). Conversely, excessively strong metal‐oxygen interactions typically promote the cleavage of the O─O bond, thereby hindering the formation of the crucial ^*^OOH intermediate required for efficient H_2_O_2_ production.

The adsorption‐free energy of *OOH (ΔG_*__OOH_) is a crucial indicator for assessing catalytic activity in the ORR, with the most favorable active sites typically located at the summit of the volcano plot.^[^ ^25^ ^]^ As displayed in Figure 5d, the ΔG_*__OOH_ values of Fe and C sites in Fe_3_O_4_(111)‐gra and Fe_3_O_4_(220)‐gra are close to the volcano plot peak compared with Fe_3_O_4_(111), Fe_3_O_4_(220) and Graphite. This observation indicates that heterostructure with Fe_3_O_4_ and graphene effectively lowers the reaction barrier for the electrochemical reduction of O_2_ to H_2_O_2_, thereby enhancing the reaction kinetic at both Fe and C active sites. Specifically, Fe_3_O_4_(111)‐gra prefers to generate H_2_O_2_ at lower potentials via the 2e^−^ pathway (Figure 5e and Figure S24, Supporting Information). In addition, the weak adsorption of oxygen (ΔG_*__O_) favors H_2_O_2_ formation, with ΔG_*__O_‐ΔG_*__OOH_ serving as a descriptor that links H_2_O_2_ selectivity to the active sites.^[^ ^17^, ^26^ ^]^ Fe_3_O_4_(111)‐gra exhibits the strongest tendency to suppress H_2_O formation, making it the most promising catalyst for driving H_2_O_2_ production (Figure 5f). Indeed, these predictions are in excellent agreement with our experimental results, where the heterojunction formed by Fe_3_O_4_ and carbon‐based materials optimizes the electronic distribution, regulates the adsorption of the *OOH intermediate on the Fe and C active sites while favoring the preservation of the O─O bond, thereby triggering high 2e^−^ activity at multiple active centers (Figure 5g).

## 3 Conclusion

In summary, an environmentally compatible Fe‐based deep eutectic solvent (PEG200/FeCl_3_·6H_2_O‐DES) was designed to serve as an all‐in‐one platform that supplies a carbon and an iron source. The hierarchically porous tremella‐like heterojunction formed by ultrafine cubic Fe_3_O_4_ nanoparticles within the amorphous carbon (UFe_3_O_4_@HPAC) was prepared using PEG200/FeCl_3_·6H_2_O‐DES via a two‐step annealing process, exhibiting remarkable overall and intrinsic 2e^−^ ORR activity. In brief, a high H_2_O_2_ selectivity of 96% and an impressive turnover frequency (TOF) of 67.5 s^−1^ at 0.6 V vs RHE was obtained in UFe_3_O_4_@HPAC. Moreover, the UFe_3_O_4_@HPAC electrode showed remarkable stability for 100 h and a high H_2_O_2_ production rate of 8.1 g^−1^ L^−1^ h^−1^, which can provide the H_2_O_2_ suitable for various medical applications (3.0–7.8 wt%). Additionally, integrating on‐site H_2_O_2_ production with the electro‐Fenton process enabled the rapid decomposition of contaminants. Theoretical studies and electrochemical analyses reveal that the unique heterostructure, with the synergistic effect between Fe_3_O_4_ and the carbon matrix, optimizes the electronic structure, enhancing electronic conductivity. The electronic rearrangement in UFe_3_O_4_@HPAC stabilizes the *OOH intermediate while preventing the cleavage of the O─O bond, thereby triggering thermodynamically advantageous active sites for the 2e^−^ ORR. This study provides new pathways and insights for designing transition metal‐based catalysts with controllable morphology and phase composition for efficient oxygen electrolysis.

## Conflict of Interest

The authors declare no conflict of interest.

## Supporting information



Supporting Information

## Data Availability

The data that support the findings of this study are available from the corresponding author upon reasonable request.
