# Supplementary material for: Heterostructure of Fe3O4 Confined in Hierarchical Porous Carbon for Interface‐Enhanced Medical‐Grade H2O2 Electrosynthesis
Source: Adv Sci (Weinh). 2025 May 19;12(27):2502388. doi: 10.1002/advs.202502388 (PMC12279173; doi:10.1002/advs.202502388)
Supplement: Supplementary file 1 — Supporting Information [file ADVS-12-2502388-s001.docx]

Supporting Information

Heterostructure of Fe_3_O_4_ Confined in Hierarchical Porous Carbon for Interface-Enhanced Medical-Grade H_2_O_2_ Electrosynthesis

Yanan Shi, Li-Li Zhang, Chongyang Wang, Shaohui Sun^*^

Yanan Shi, Chongyang Wang, Shaohui Sun

Engineering Research Center of Advanced Functional Material Manufacturing of Ministry of Education, School of Chemical Engineering, Zhengzhou University, Zhengzhou 450001, China; State Key Laboratory of Coking Coal Resources Green Exploitation, Zhengzhou University, Zhengzhou 450001, China
E-mail: shaohui99@zzu.edu.cn

Li-Li Zhang
Interdisciplinary Research Center for Sustainable Energy Science and Engineering (IRC4SE^2^), School of Chemical Engineering, Zhengzhou University, Zhengzhou 450001, China

**Experimental Section**

**Materials**

Iron(III) chloride hexahydrate (FeCl_3_·6H_2_O), Polyethylene glycol (PEG200), potassium thiocyanate (KSCN), cerium(IV) sulfate (Se(SO_4_)_2_), Ammonium bicarbonate (NH_4_HCO_3_), and polytetrafluoroethylene preparation (PTFE, 60 wt %) were purchased from Aladdin Co., Ltd. Carbon black (CB) was obtained from Cabot Corporation Co., Ltd. Potassium hydroxide (KOH) was sourced from Tianjin Fengchuan Chemical Reagent Technology Co., Ltd. Isopropanol was supplied by Sinopharm Chemical Reagent Co., Ltd. Nafion perfluorinated resin solution (5 wt%) was purchased from Shanghai Yien Chemical Technology Co., Ltd. All chemical reagents were used as received without further purification.

**Synthesis of PEG200/FeCl_3_·6H_2_O-DES**

FeCl_3_·6H_2_O and PEG200 were mixed at a molar ratio of 0.44:1 and heated at 50 °C with stirring for 3 h until a uniform and transparent liquid was formed. The resulting mixture was dried under vacuum at 60 °C overnight, yielding the deep eutectic solvent PEG200/FeCl_3_·6H_2_O-DES.

**Synthesis of UFe_3_O_4_@HPAC**

The material was prepared via a two-step thermal treatment. PEG200/FeCl_3_·6H_2_O-DES was introduced into a ceramic boat and positioned at the center of a tube furnace. After purging with N_2_, the sample underwent pyrolysis at 220°C for 3 h under N_2_. The product was ground into a black powder, then washed with 50 mL water-ethanol mixture (V_water_ : V_ethanol_ = 3:1). This washing procedure was repeated four times and then dried at 80°C for 12 h. The final annealing step was carried out at 550°C for 3 h under flowing N_2_, resulting in the formation of UFe_3_O_4_@HPAC.

**Synthesis of Fe_3_O_4_**

FeCl_3_·6H_2_O was thermally decomposed via a single-step annealing at 550°C for 3 hours to obtain Fe_3_O_4_.

**Synthesis of HPAC**

UFe_3_O_4_@HPAC was first reduced for 3 h at 550°C in an H_2_ atmosphere. Subsequently, the sample was dispersed in 250 mL of 2 M H_2_SO_4_ and refluxed at 80°C under continuous stirring for 24 h. Finally, the sample was repeatedly washed with deionized water until neutral pH was reached, then dried overnight at 80°C in a drying oven to obtain HPAC.

**Characterization**

Powder X-ray diffraction (XRD) experiments were performed on a D8 Advance, and the source was a Cu-K_α1_ radiation at 40 kV and 40 mA. Transmission electron microscopy (TEM), High-resolution TEM (HRTEM), high-angle annular dark-field scanning TEM (HAADF-STEM) and Energy-dispersive X-ray mapping (EDX) were carried out on a JEOL JEM-F200 field emission electron microscope at 200 kV. Nitrogen adsorption-desorption measurements were conducted at 77 K using a Micromeritics ASAP 2460 Plus system. X-ray photoelectron spectroscopy (XPS) analyses were performed on an AXIS SUPRA 250Xi electron spectrometer utilizing an Al Kα source, with binding energies referenced to the C 1s peak at 284.8 eV. Raman spectra were obtained on a Lab RAM HR Evolution spectrometer with a 532 nm laser. Fourier transform infrared (FT-IR) spectra were recorded on a Bruker Tensor II infrared spectrometer. The contact angle was measured at room temperature using a goniometer (Krüss DSA100, Germany) via the sessile drop method, with values averaged over at least three independent measurements.

**Electrochemical measurements**

Electrochemical measurements were conducted at room temperature using a CHI 760E electrochemical workstation equipped with an RRDE-3A rotating ring-disk electrode setup and a three-electrode configuration. A platinum (Pt) sheet served as the counter electrode, and an Ag/AgCl electrode was used as the reference electrode. Measurements were conducted in 0.1 M KOH solution. The rotating ring-disk electrode (RRDE) with a glassy carbon electrode (geometric area: 0.1256 cm^−2^) and a platinum ring electrode (geometric area: 0.1885 cm^−2^) was used as the working electrode. All potentials were converted to the reversible hydrogen electrode (RHE) scale using the Nernst equation E_RHE_ = E_Ag/AgCl_ + 0.197 +0.0591 × pH. Catalyst ink was prepared by dispersing 2.0 mg of catalyst in a mixture of 0.48 mL deionized water, 0.49 mL isopropyl alcohol, and 30 µL Nafion solution (5 wt%), followed by ultrasonication for 40 min to ensure homogeneity. A 5 µL aliquot of the catalyst ink was then drop-cast onto the RRDE disk electrode and allowed to dry at room temperature, forming a uniform thin-film electrode with a catalyst loading of 80 µg cm^−2^. Before measurements, all catalysts were activated electrochemically via cyclic voltammetry (CV) at a scan rate of 50 mV s^−1^ until stable voltammograms were obtained. Linear sweep voltammetry (LSV) curves were conducted under O_2_-saturated at a scan rate of 5 mV s^−1^ with a rotation speed of 1600 rpm. The potential of the Pt ring electrode was maintained at 1.2 V vs. RHE to monitor electrogenerated H_2_O_2_ on the disk electrode. The H_2_O_2_ selectivity and electron transfer number (*n*) were calculated according to the following equations:

$\text{H}_{\text{2}}\text{O}_{\text{2}}\text{ selectivity (\%) =200 × }\frac{{\text{I}_{\text{R}}}/\text{N}}{\text{I}_{\text{D}}\text{ + }{\text{I}_{\text{R}}}/\text{N}}$ (1)

$\text{n}\text{ = 4 × }\frac{\text{I}_{\text{D}}}{\text{I}_{\text{D}}\text{-}{\text{I}_{\text{R}}}/\text{N}}$ (2)

where *I*_R_ and *I*_D_ are the ring current and disk current. N is the current collection efficiency of the ring electrode.

Cyclic voltammetry (CV) was measured at various scan rates to estimate the electrochemical active surface area (ECSA). The CV curves were recorded in 0.1 M KOH over a potential range where Faradaic reactions are negligible, with scan rates (ν) of 40, 60, 80, 100, 120, 140 mV s^−1^. The capacitance of the double layer (*C*_dl_) was calculated from the slope of the plot of average current density versus scan rate:

$\text{C}_{\text{dl}}\text{ = }\frac{\text{j}}{\nu}$ (5)

Where *j* is the average current density, determined from the CV curves. The ECSA was estimated based on *C*_dl_, as there a direct proportional relationship between them:

$\text{ECSA = }\frac{\text{C}_{\text{dl}}\text{ × }\text{A}_{\text{geom}}}{\text{C}_{\text{s}} \times m_{cat}}$ (6)

where C_s_ is the specific capacitance, and m_cat_ is the catalyst loading on the disk electrode.

The EIS measurements were conducted by applying an AC voltage with 5 mV amplitude in a frequency range from 0.01-10^6^ Hz, measured at 0.7 V vs. RHE in 0.1 M KOH.

To gather additional information on active sites, a poisoning test was conducted on the samples. A specific amount of KSCN was added to the electrolyte solution to obtain a concentration of 0.1 M KOH with 10 mM KSCN. Additionally, a test for H_2_O_2_ decomposition was carried out on various catalysts in 0.1 M KOH with 10 mM H_2_O_2_.

The kinetic current density (*j*_K_) was determined using the Koutecky-Levich (K-L) equation:

$\frac{\text{1}}{\text{j}}\text{ = }\frac{\text{1}}{\text{j}_{\text{L}}}\text{ + }\frac{\text{1}}{\text{j}_{\text{K}}}\text{= }\frac{\text{1}}{\text{B}\text{ × }\text{ω}^{\text{1/2}}}\text{ + }\frac{\text{1}}{\text{j}_{\text{K}}}$ (7)

$\text{B}\text{ = 0.62}\text{n}\text{F}\text{C}_{\text{o}}\text{D}_{\text{o}}^{\text{3/2}}\nu^{\text{-1/6}}$ (8)

Where *j* is the measured current density, *j*_K_ is the kinetic current density, and *j*_L_ is the diffusion-limiting current density. ω represents the angular velocity of the disk (rad s^-1^), *n* is the number of electrons transferred in the ORR, and F is the Faraday constant (96485 C mol^−1^). C_o_ and D_o_ denote the bulk concentration of O_2_ (0.84 × 10^−3^ mol cm^−3^) and diffusion coefficient of O_2_ (1.65 × 10^−5^ cm^2^ s^−1^), respectively. *ν* is the kinematic viscosity of the electrolyte (0.01 cm^2^ s^−1^). All parameters are determined under standard atmospheric pressure at 25 °C.

Mass Activity and Turnover Frequency (TOF) were calculated as follow. The mass activity (mA mg^−1^) values were calculated from the catalyst mass m (mg cm^−2^) and the kinetic current density *j_K_* (mA cm^−2^):

$\text{Mass }\text{activity}\text{ }{\text{(}\text{H}}_{\text{2}}\text{O}_{\text{2}}\text{) }\text{= }\frac{\text{j}_{\text{K}}\text{ × }\text{A}_{\text{geom}}\text{ }\text{×}\text{ }\text{r}_{\text{f}}}{m_{cat}} \text{×}\text{ }\text{H}_{\text{2}}\text{O}_{\text{2}}\text{ selectivity}$ (6)

Where *r*_f_ is the roughness factor, defined as the ratio of the actual surface area of the electrode to its geometric surface area. The TOF values were calculated using the following equation:

$\text{TOF}\text{ }\text{= }\frac{\text{j}_{H2O2}\text{ × }\text{A}_{\text{geom}}}{2\text{×}F{\text{×}n}_{\mathrm{Fe}}}$ (6)

Where A_geom_ is the surface area of the disk electrode, the number 2 represents 2 electrons per mole of O_2_, F is the Faraday constant (96485 C mol^−1^), *n*_Fe_ is the moles of Fe atom coated on the electrode calculated from relative element contents in the XPS spectra, and *j*_H2O2_ is the H_2_O_2_ current density calculated using the following equation:

$\text{j}_{\text{H2O2}}\text{ = }\frac{\text{I}_{\text{R}}}{\text{N ×}\text{ A}_{\text{geom}}}$ (3)

The accelerated degradation test (ADT) was performed in O_2_-saturated 0.1 M KOH, involving 5000 cycles of cyclic voltammetry (CV) within a voltage range of 0−1.2 V vs. RHE. LSV curves were recorded before and after the CV cycling.

**Practical Device Assembly and Measurement**

A flow cell was used to simulate a practical device, comprising a gas chamber, a cathode chamber and an anode chamber. The gas chamber was continuously purged with high-purity O_2_ under a flow rate of approximately 5 mL min^−1^. The cathode and anode chambers were circulated with a mixed electrolyte containing 0.5 M Na_2_SO_4_ and 0.1 M KOH at a flow rate of 0.5 mL min^−1^. The anode and cathode chambers were separated by a Nafion 117 membrane.

A Pt wire served as the anode. The cathode was prepared with a gas diffusion electrode (GDE) consisting of three parts: the gas diffusion layer (GDL), the support layer, and the catalyst layer. The preparation process is as follows: (1) Pretreatment of the support layer: stainless steel mesh (40-mesh) was cut into 5 cm× 5 cm pieces, soaked in ethanol, and subjected to ultrasonic cleaning for 30 minutes. After soaking, the mesh was thoroughly rinsed with deionized water and dried for subsequent use. (2) Preparation of the GDL: CB, GP, NH_4_HCO_3_, and PTFE were mixed in ethanol at a mass ratio of 3:3:1:3. The mixture was stirred vigorously at 60 °C until a paste formed. The paste was applied evenly to both sides of the pretreated stainless-steel mesh. The coated mesh was then pressed in a mold at 8−10 MPa for 20 min. The sample was pyrolyzed in a muffle furnace at 330 °C for 1 h, with a heating rate of 1 °C min^-1^, to produce the blank GDL. (3) Loading of the Catalyst Layer: A catalyst ink was prepared by dispersing 15 mg of catalyst, 5 mg of NH₄HCO₃, and 0.045 g of PTFE in 1 mL absolute ethanol, followed by ultrasonication for 40 mins to ensure homogeneity. The catalyst ink was then drop-coated onto the blank GDL and pyrolyzed in a muffle furnace at 330 °C for 1 h with a heating rate of 1 °C min^-1^ to obtain the working electro for the flow-cell.

Oxygen electrolysis was performed using a constant current from a direct current (DC) power supply. The concentration of H_2_O_2_ was determined using a cerium sulfate Ce(SO4)_2_ titration technique. In this method, yellow Ce^4+^ is reduced by H_2_O_2_ to colorless Ce^3+^ (2Ce^4+^ + H_2_O_2_ = 2Ce^3+^ + 2H+ + O_2_). The yield of H_2_O_2_ is calculated based on the consumption of Ce^4+^ using the following equation:

$\text{C}_{\text{H}_{\text{2}}\text{O}_{\text{2}}}\text{ = }\frac{\text{V}_{\text{Ce}^{\text{4+}}}\text{ × }\text{C}_{\text{Ce}_{\text{before}}^{\text{4+}}}\text{ - }\left( \text{V}_{\text{Ce}^{\text{4+}}}\text{ +}{\text{ }\text{V}}_{\text{electrolyte}} \right)\text{× }\text{C}_{\text{Ce}_{\text{after}}^{\text{4+}}}}{\text{2 × }\text{V}_{\text{electrolyte}}}$ (9)

where *V*_Ce_^4+^ is the volume of added Ce(SO4)_2_, *C*_Cebefore_^4+^ and *C*_Ceafter_^4+^ are the concentration of Ce^4+^ before and after reaction, measured at a wavelength of 320 nm using ultraviolet-visible (UV-vis) spectroscopy, and V_electrolyte_ is the volume of the electrolyte solution. The faradaic efficiency (FE) was calculated using the following equation:

$\text{FE(\%) = }\frac{\text{2 ×C ×V ×F}}{\text{Q}}\text{ ×100\%}$ (10)

where F is the Faraday constant (96485 C mol^-1^), C is the concentration of generated H_2_O_2_, V is the volume of the electrolyte (30 mL), and Q is the total charge passed during the electrolysis. A standard Ce(SO4)_2_ solution (0.5 mM) was prepared by dissolving Ce(SO4)_2_ salts in 0.5 M sulfuric acid. Calibration curves between absorbance and Ce^4+^ concentration were determined by measuring the absorbance at 320 nm of different Ce(SO4)_2_ solutions with known concentrations (0.01–0.5 mM) (*y* = 5.315*x* – 0.037; R^2^ = 0.99995). [1]

**Applied for electro-Fenton process**

To further explore the in-situ application of the catalyst for the direct electrochemical synthesis of H_2_O_2_, an electro-Fenton experiment for pollutant degradation was conducted. The experiment was performed in a custom-built one-compartment cell. The GDE with an active area of 7.5 cm^2^ served as the working electrode. The model contaminants included 12 mL of 500 mg L^−1^ Rhodamine B (RhB) and 500 mg L^−1^ methylene blue (MB). The Fenton reagent used consisted of 0.5 mM FeSO_4_, 0.05 M Na_2_SO_4_ and 3 M H_2_SO_4_, which were used as the electrolyte. A constant current was applied to the cell during the E-Fenton process, with solutions replaced after complete pollutant degradation. The degradation rates of RhB and MB was determined by UV-visible spectrophotometry. The calibration curves for RhB and MB concentrations were reported in a recent publication.

**Computational details**

All the density functional theory (DFT) calculations were performed using the Vienna ab-initio simulation package (VASP). [2] The exchange-correlation interactions were described by the generalized gradient approximation (GGA) in the form of the Perdew-Burke-Ernzerhof of functional (PBE). [3] A plane-wave cutoff energy was set to 400 eV, the convergence threshold was 10^−5^ eV, and −0.02 eV/Å for energy and force, respectively. A Monkhorst-Pack 4×4×1 k-point grid was used to sample the Brillouin zone. A 5×5 monolayer of graphitic sheet was constructed as the intrinsic model, with a vacuum space above the graphitic sheets of 15 Å. The change in Gibbs free energy (ΔG) of each adsorbed intermediate is calculated based on the computational hydrogen electrode method developed by Nørskov et al. [4] At zero potential and pH=0, the free energy G is defined as:

$\text{ΔG = }\text{Δ}\text{E + }\text{ΔE}_{\text{ZPE}}\text{ – TΔS}$ (11)

where ΔE is the energy change obtained from DFT calculation, ΔE_ZPE_ denotes the zero-point energy estimated within the harmonic approximation, TΔS is the entropy change at 298.15 K.


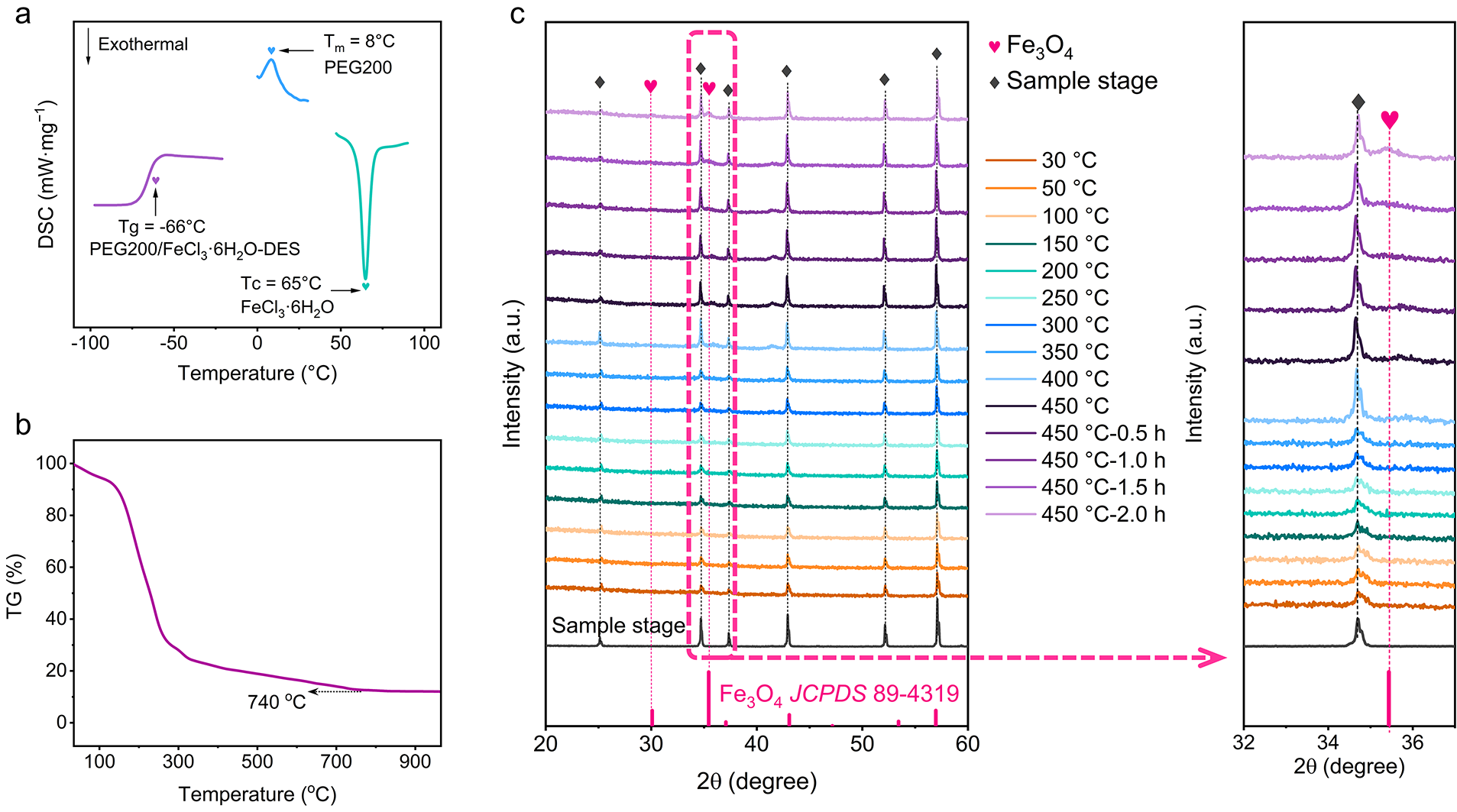


Figure S1. (a) DCS cures of PEG200, FeCl_3_·6H_2_O, and PEG200/FeCl_3_·6H_2_O-DES. (b) TG cure of PEG200/FeCl_3_·6H_2_O-DES. (c) In-suit XRD at different temperature.

Fe-based deep eutectic solvent (PEG200/FeCl_3_·6H_2_O-DES) was designed by simply heating polyethylene glycol (PEG200) with FeCl_3_·6H_2_O, serving as a multifunctional platform that supplies carbon and iron source and functions as a solvent and template. The formation of PEG200/FeCl_3_·6H_2_O-DES was verified by differential scanning calorimetry (DSC). As shown in Figure S1a, the melting point of PEG200/FeCl_3_·6H_2_O-DES (−66°C) is significantly lower than that of PEG200 (8°C) and FeCl_3_·6H_2_O (65°C), indicative of its deep eutectic behavior. the first-step annealing temperature (T1) should be below the complete decomposition temperature of PEG200/FeCl_3_·6H_2_O-DES, ideally around the temperature at which a 50% mass loss is observed in thermogravimetric analysis (Figure S1b).


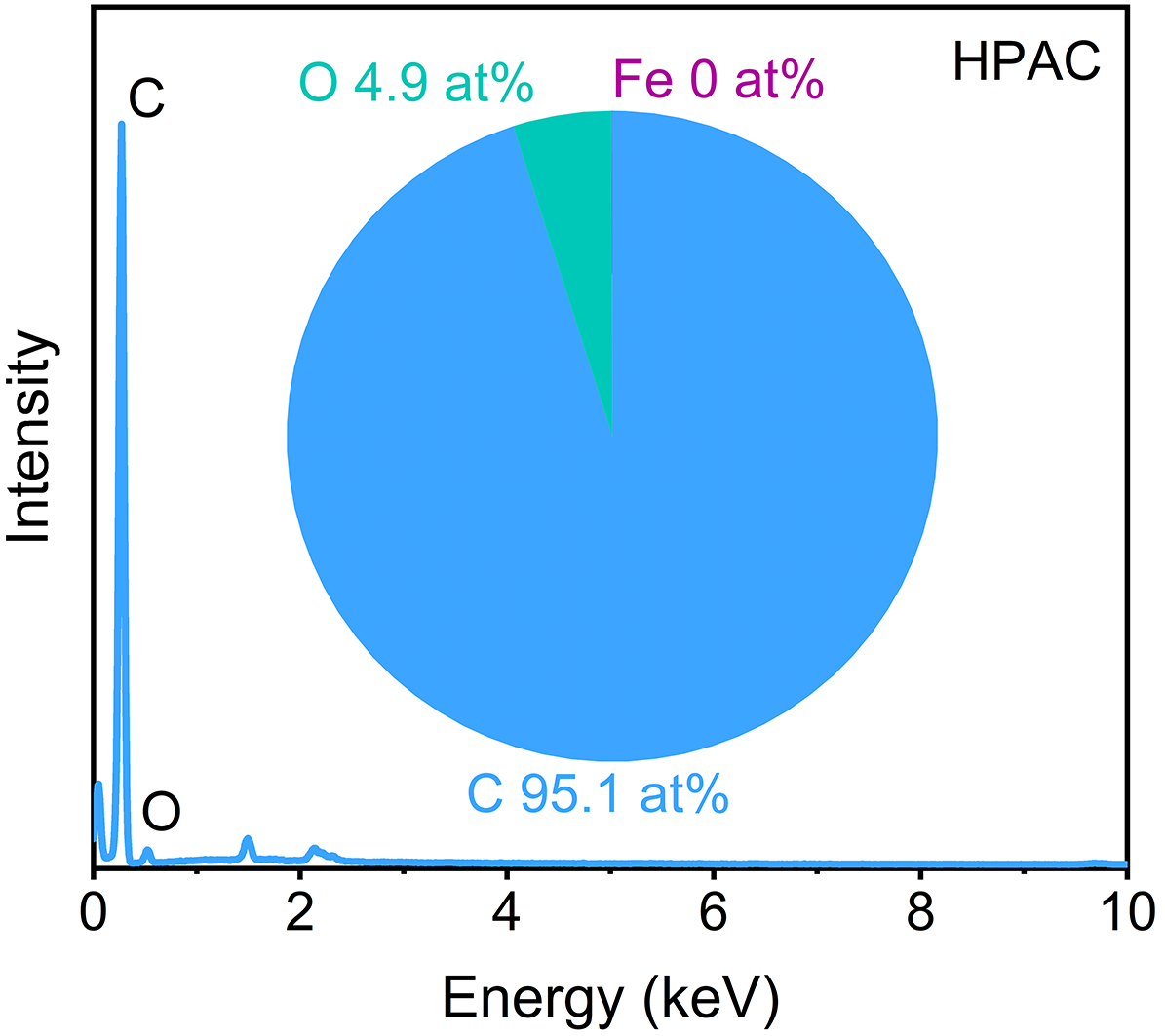


Figure S2. EDX of HPAC.


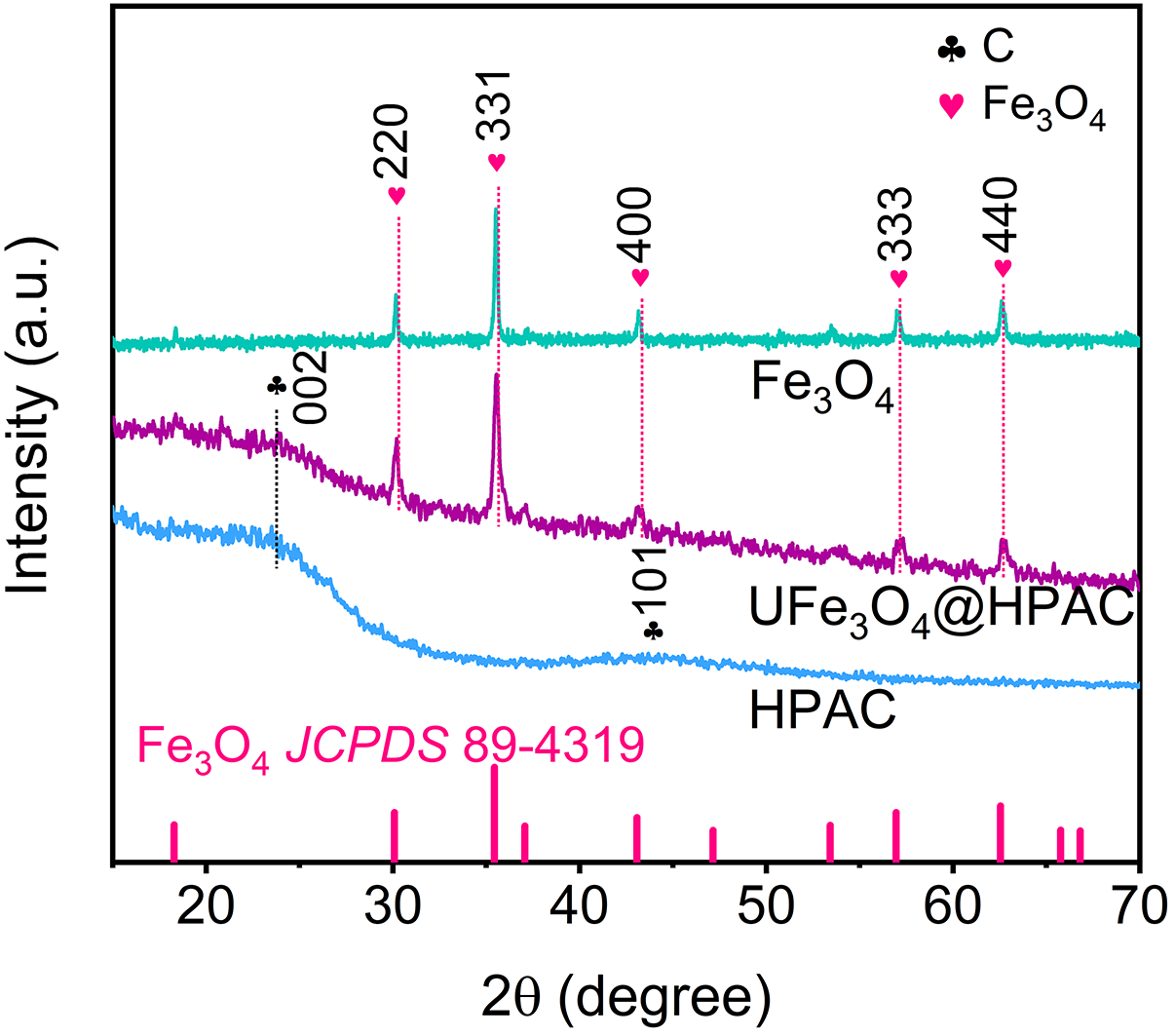


Figure S3. XRD patterns of HPAC, UFe_3_O_4_@HPAC, and Fe_3_O_4_.


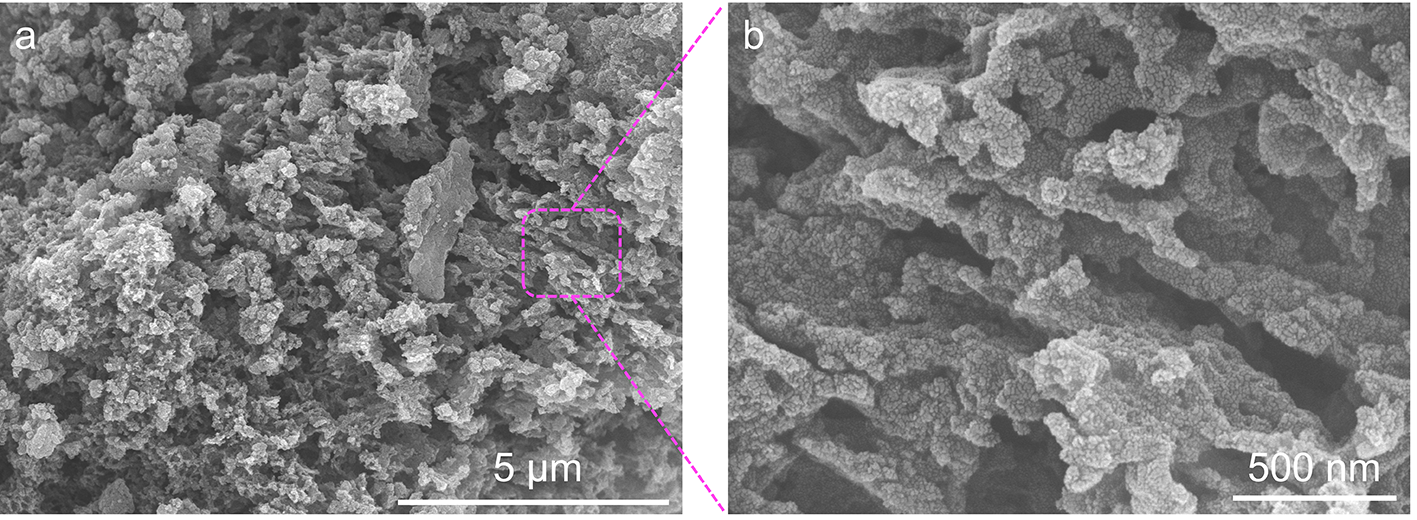


Figure S4. SEM images of UFe_3_O_4_@HPAC.


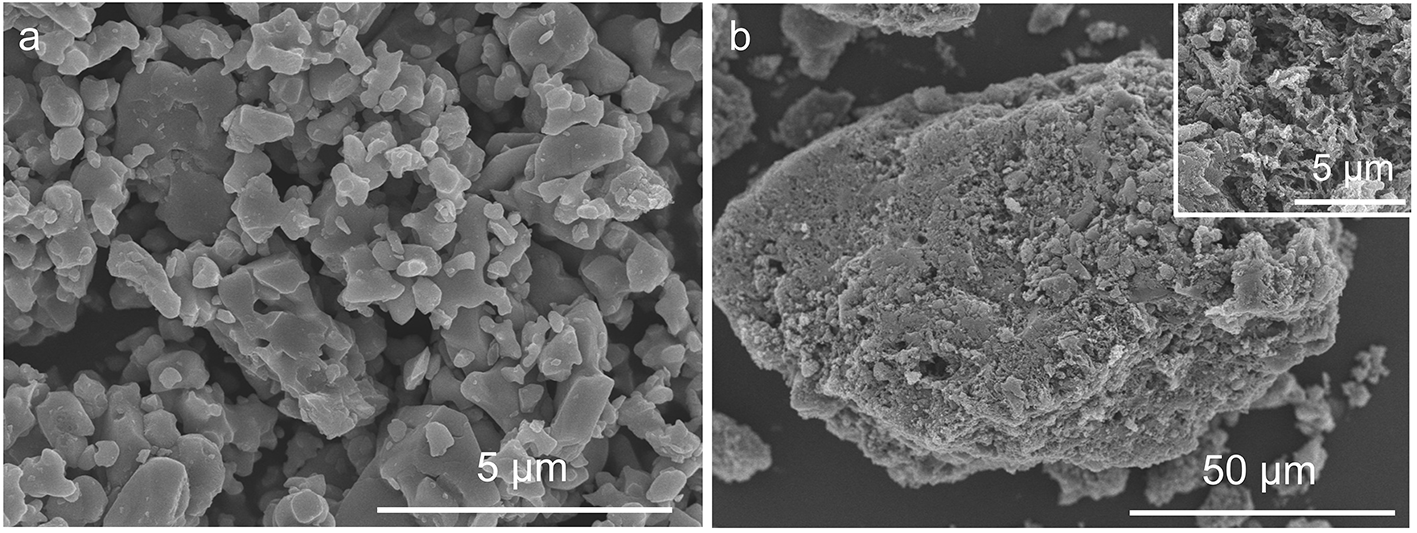


Figure S5. SEM images of (a) Fe_3_O_4_, and (b) HPAC.


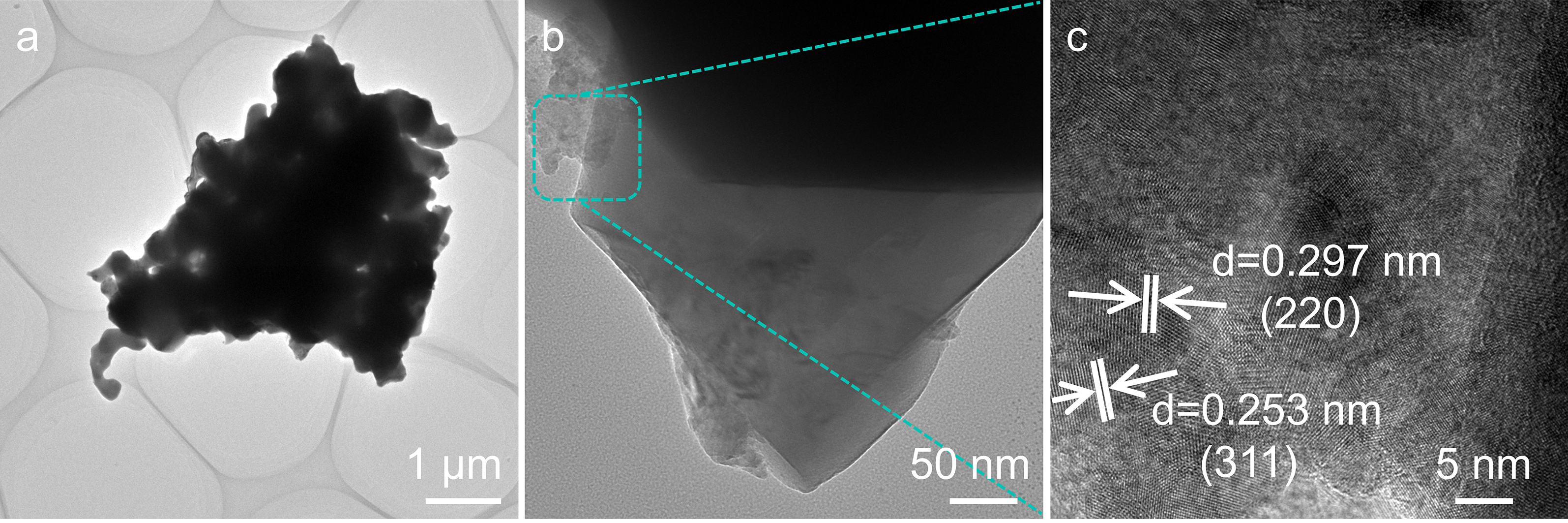


Figure S6. (a) (b) TEM and (c) HRTEM images of Fe_3_O_4_.


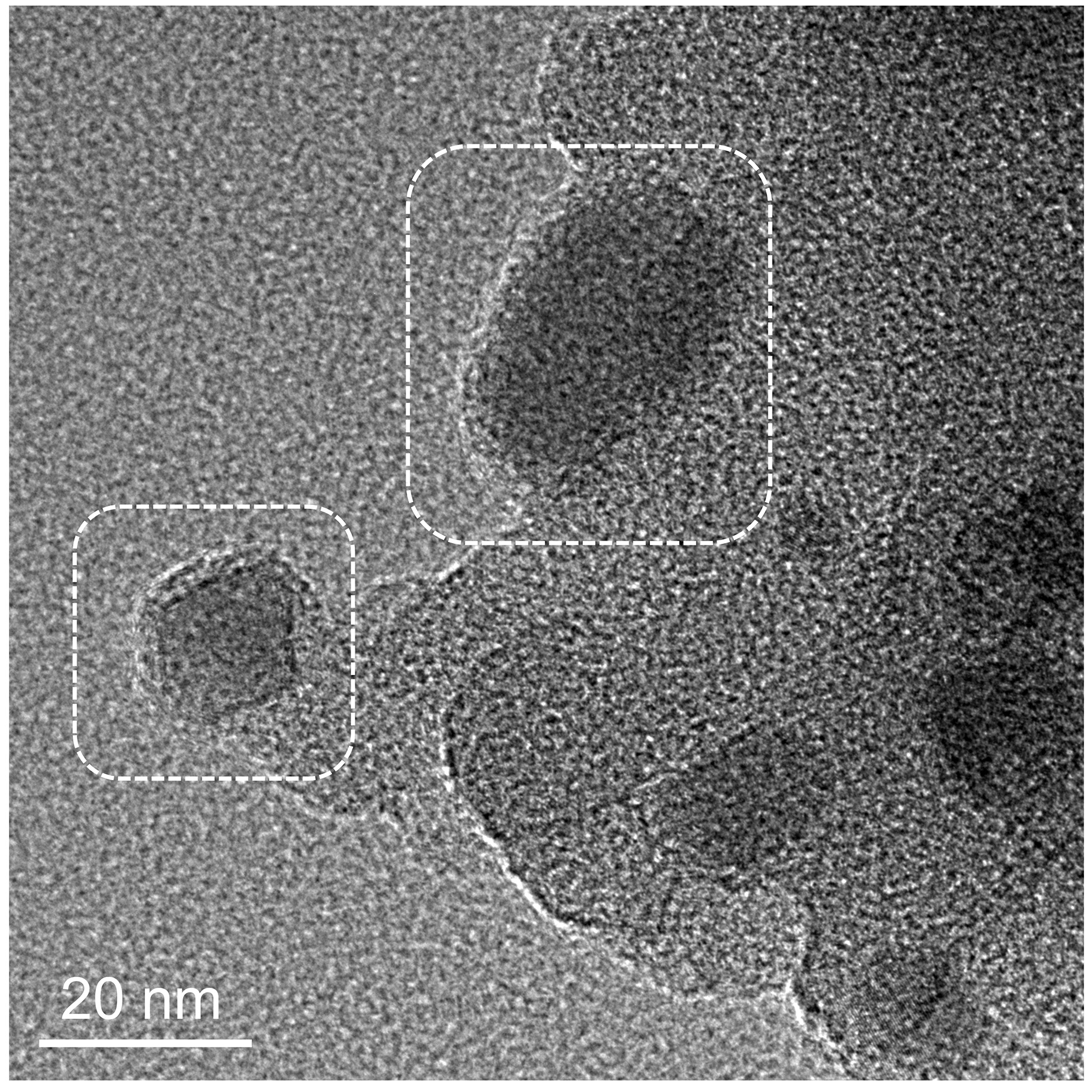


Figure S7. TEM image of UFe_3_O_4_@HPAC.

Fe_3_O_4_ nanoparticles are encapsulated or embedded within the amorphous carbon matrix.


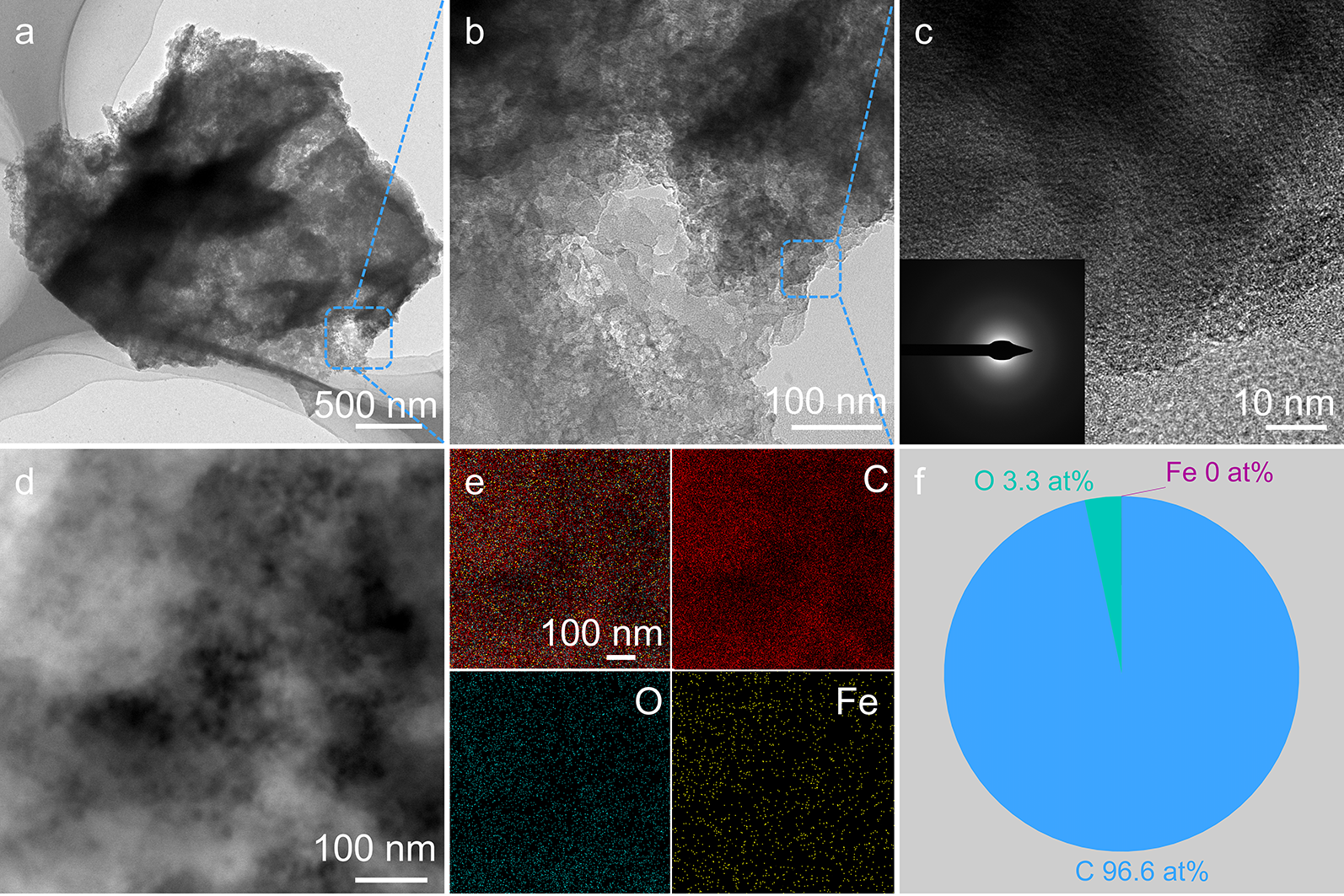


Figure S8. (a) (b) TEM, (c) HRTEM (d) HAADF-STEM images of HPAC. (e) (f) The corresponding elemental mapping of HPAC.

The TEM images reveal that the HPAC catalyst exhibits an irregular bulk morphology and consists of amorphous carbon, which aligns with XRD results. Furthermore, energy-dispersive X-ray (EDX) mapping further confirms the absence of Fe in HPAC.


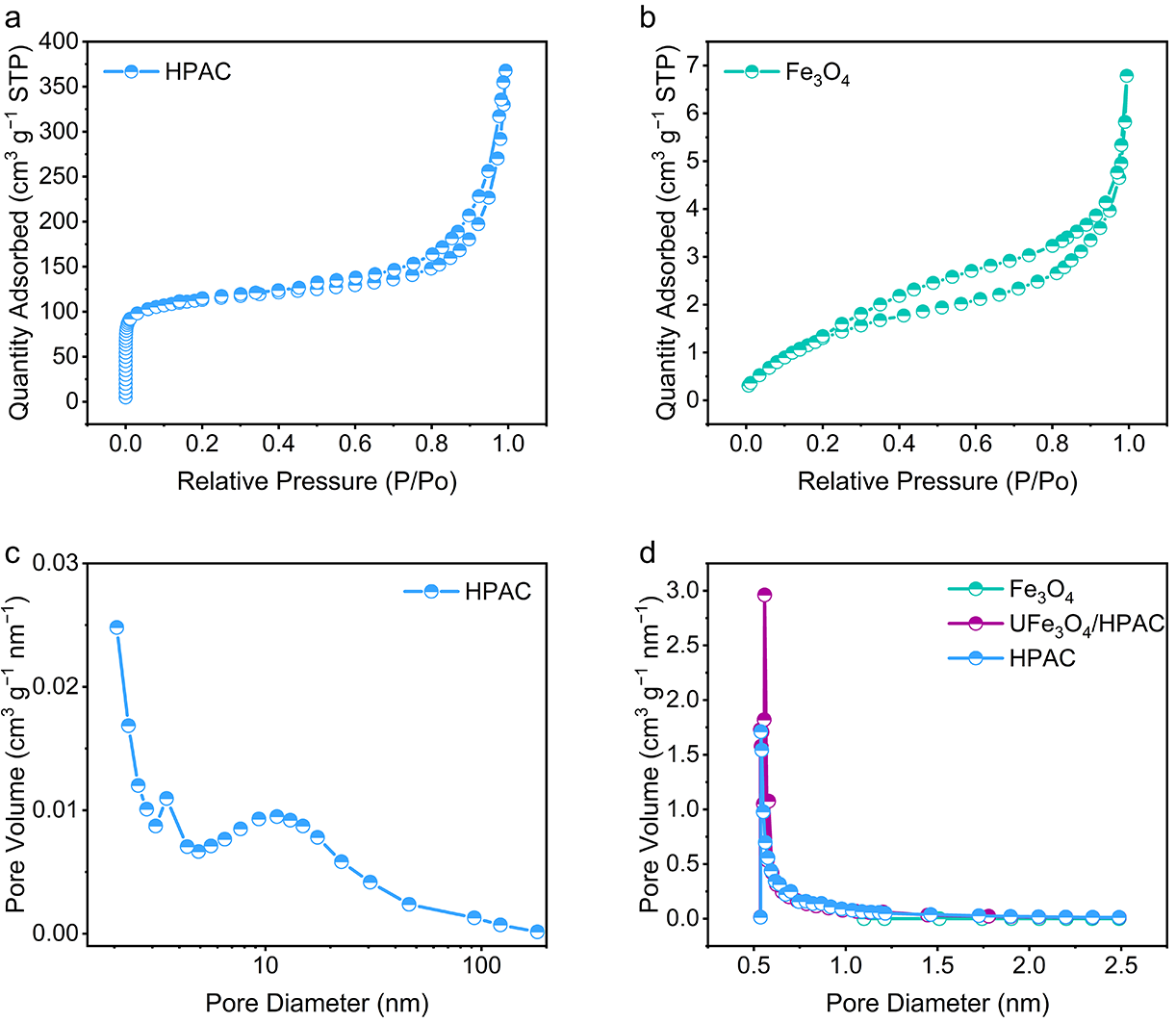


Figure S9. (a) (b) N_2_ adsorption and desorption isotherms and (c) (d) pore size distribution.


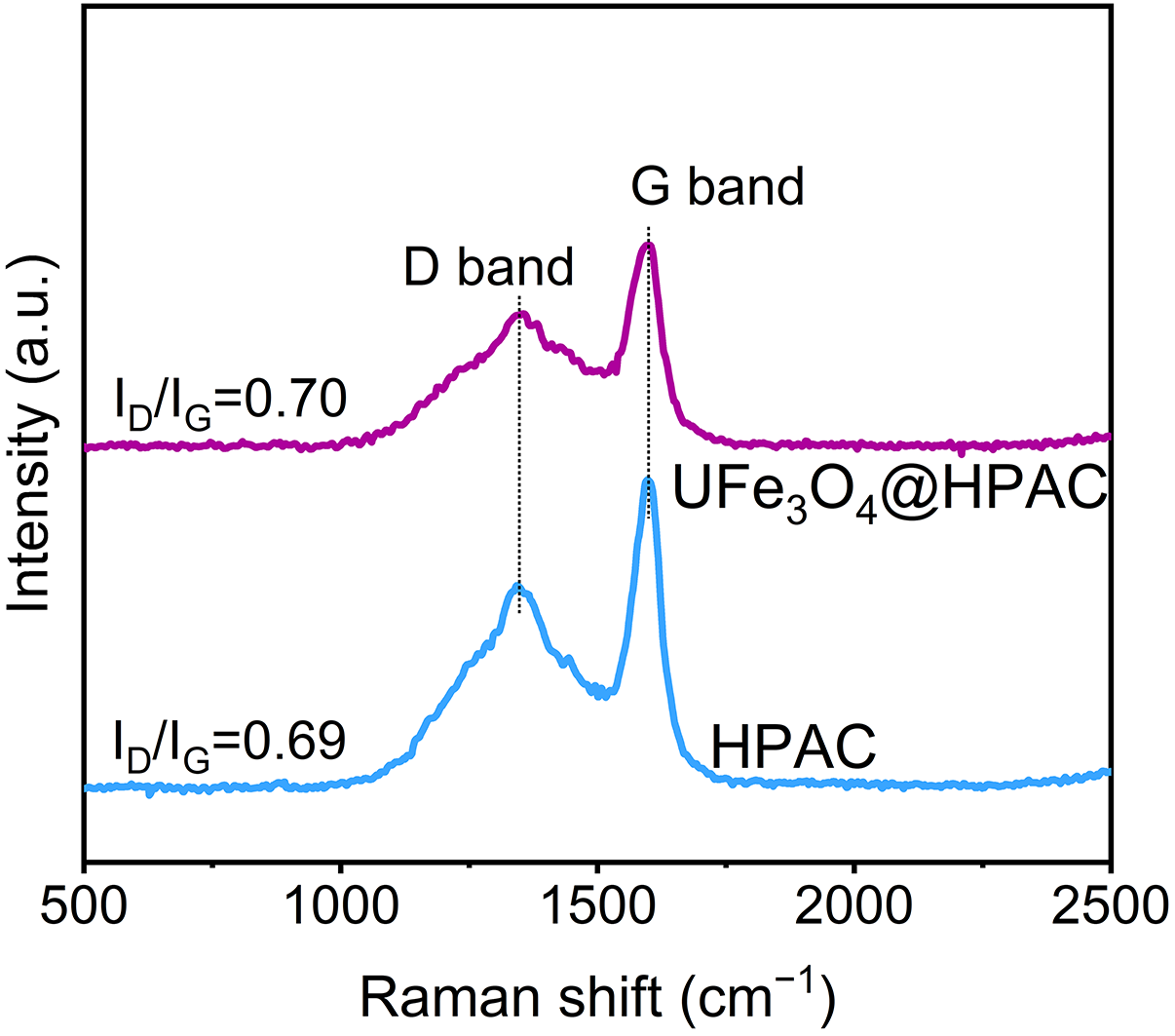


Figure S10. Raman spectra of HPAC, and UFe_3_O_4_@HPAC.


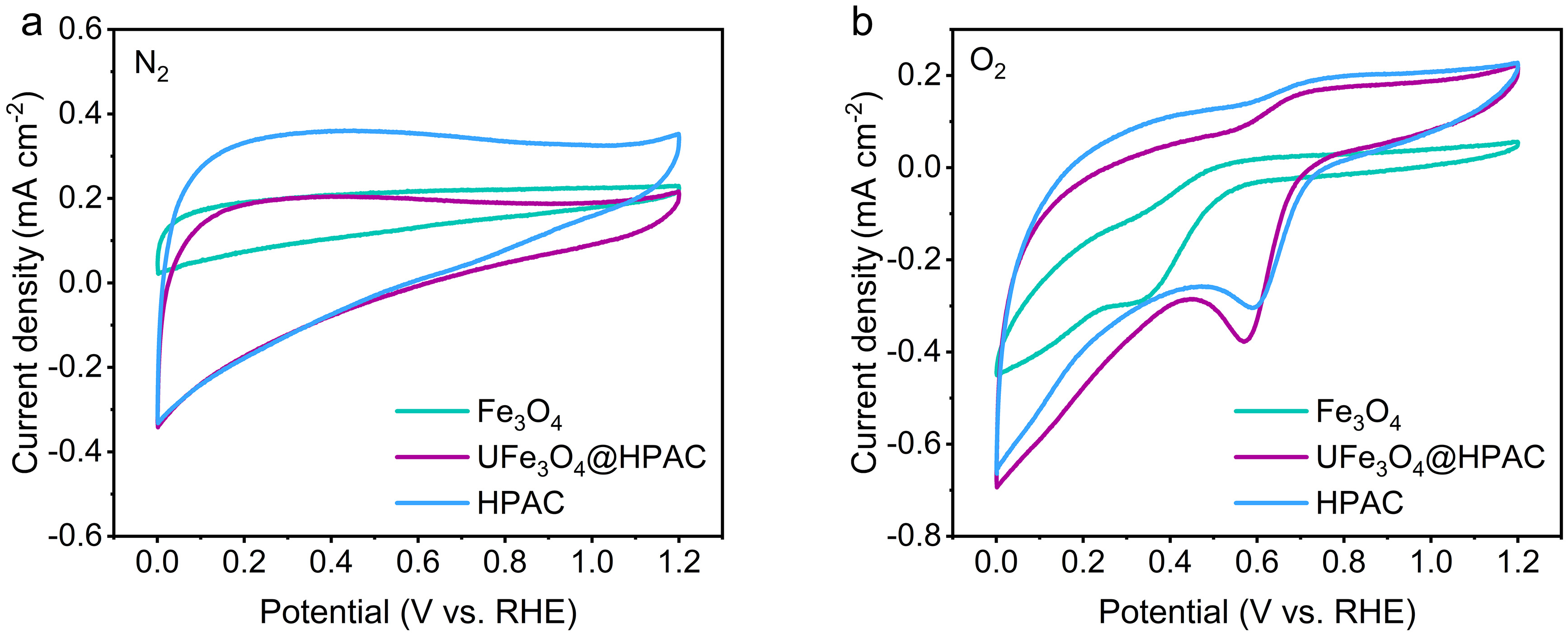


Figure S11. Cyclic voltammograms of Fe_3_O_4_, UFe_3_O_4_@HPAC, and HPAC in (a) N_2_- and (b) O_2_-saturated 0.1 M KOH.


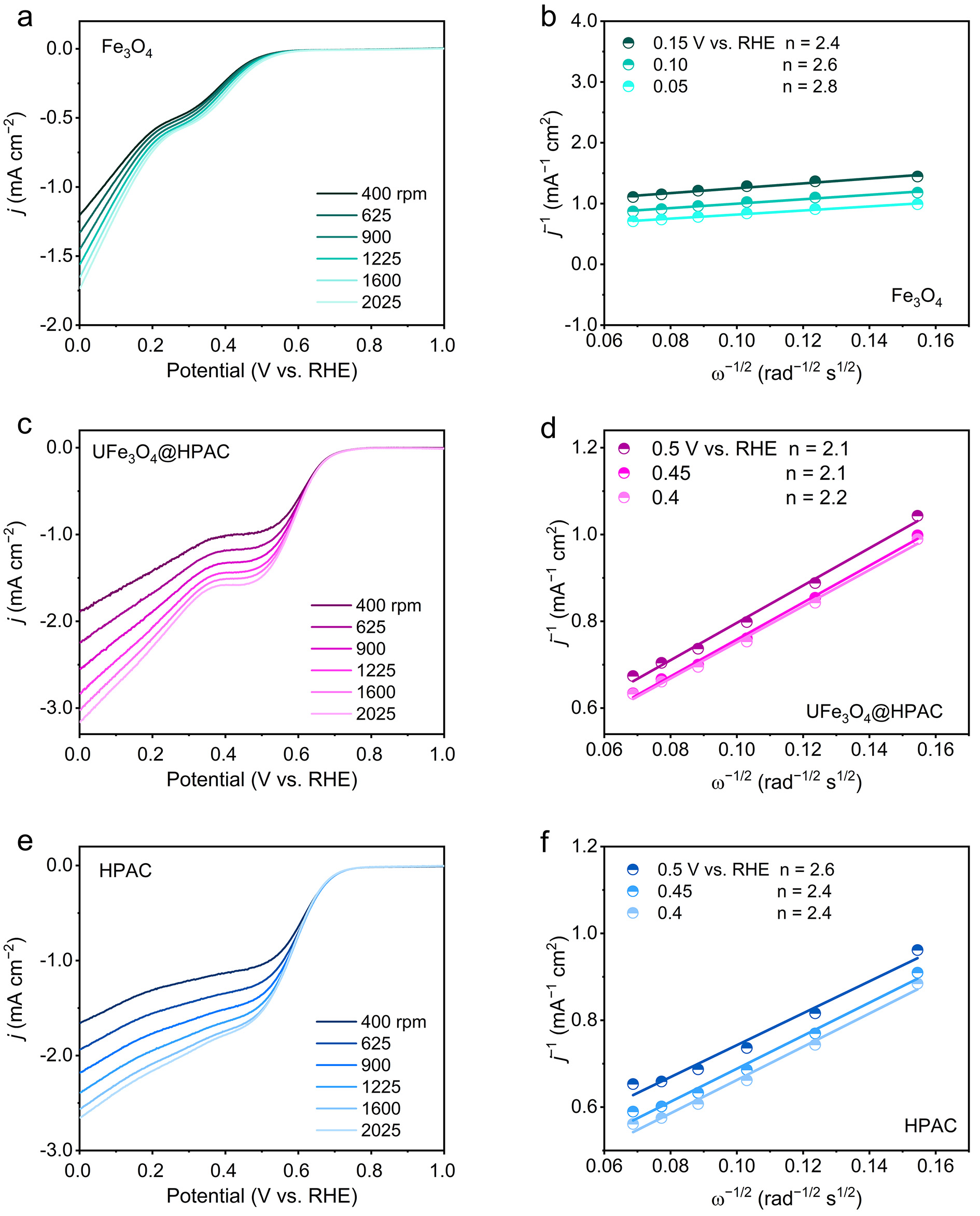


Figure S12. LSV curves of (a) Fe_3_O_4_, (c) UFe_3_O_4_@HPAC, and (e) HPAC recorded at 5 mV s^–1^ in O_2_-saturated 0.1 M KOH at various rotation speeds. Koutecky-Levich (K–L) plots of (b) Fe_3_O_4_, (d) UFe_3_O_4_@HPAC, and (f) HPAC in 0.1 M KOH at different potentials.


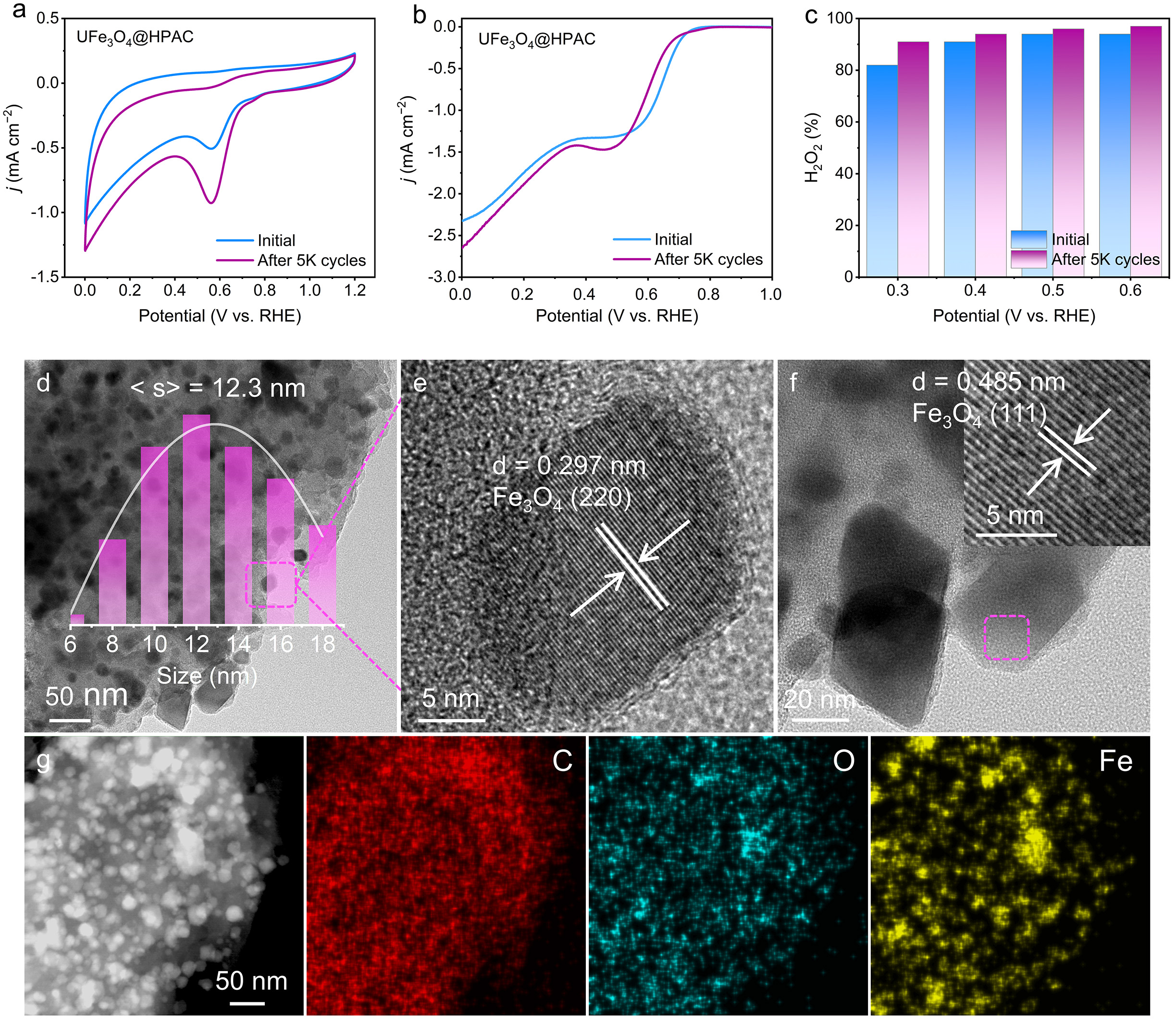


Figure S13. (a) Cyclic voltammograms, (b) LSV curves, and (c) H₂O₂ selectivity of UFe_3_O_4_@HPAC prior to and following 5,000 CV cycles during the accelerated degradation test. Post-cycling structural characterization of UFe_3_O_4_@HPAC: (d) TEM image and Fe_3_O_4_ particle size distribution, (e) (f) HRTEM images, and (g) HAADF-STEM image coupled with EDX elemental mapping.


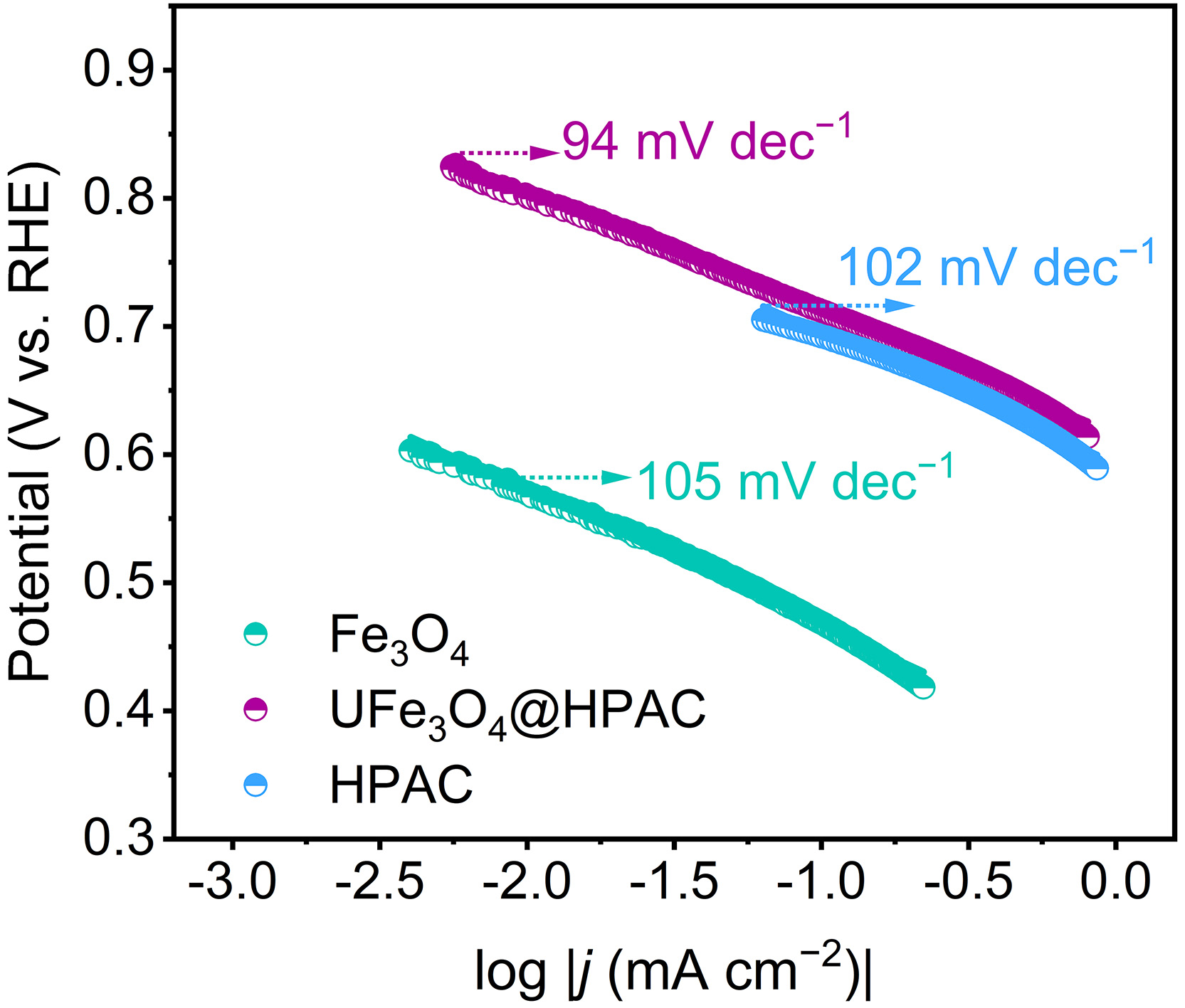


Figure S14. Tafel slopes of Fe_3_O_4_, UFe_3_O_4_@HPAC, and HPAC


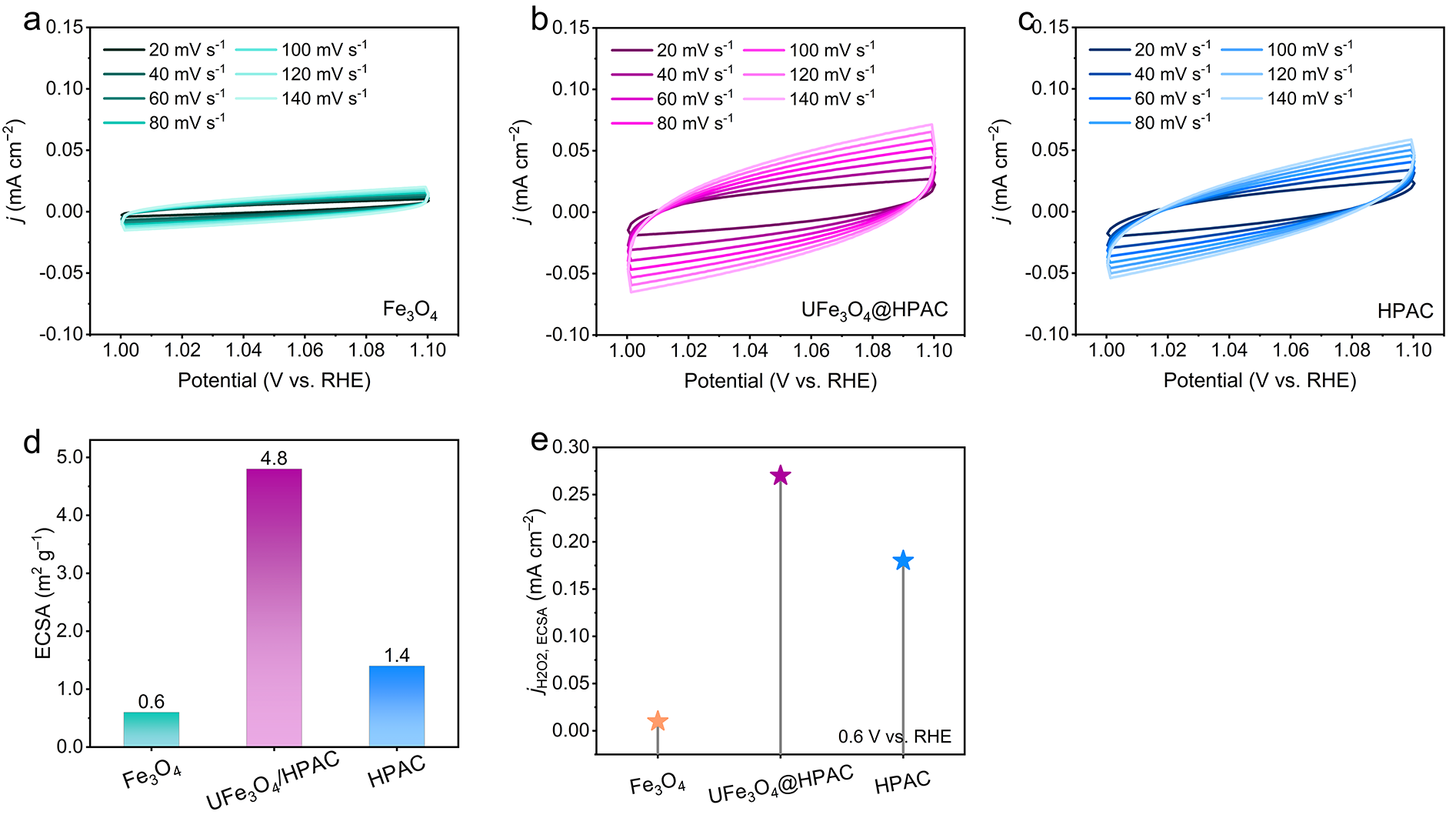


Figure S15. Cyclic voltammograms of (a) Fe_3_O_4_, (b) UFe_3_O_4_@HPAC, and (c) HPAC in 0.1 M KOH at scanning rates of 20, 40, 60, 80, 100, 120 and 140 mV s^–1^. (d) Electrochemically active surface area (ECSA) and (e) H_2_O_2_ current at 0.6 V vs. RHE normalized by ECSA (*j*_H2O2, ECSA_).


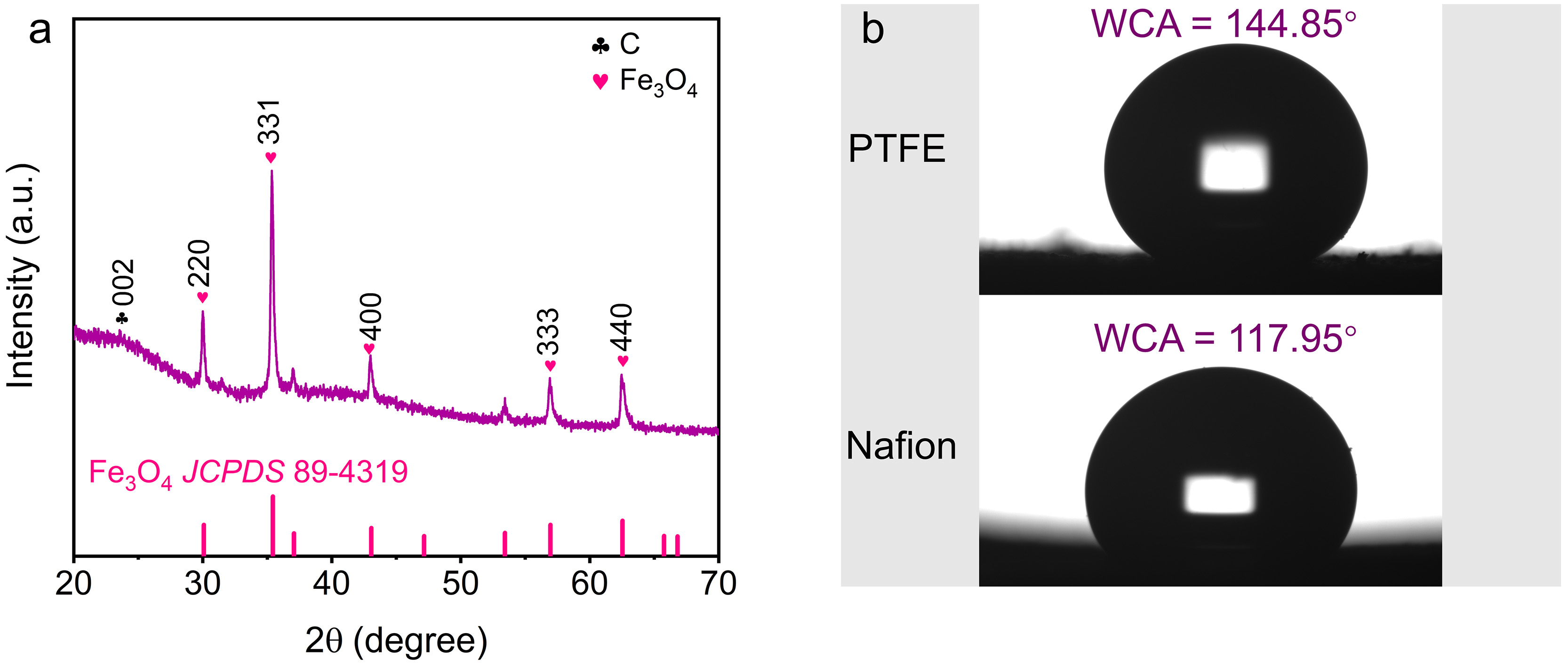


Figure S16. (a) XRD pattern for the UFe_3_O_4_@HPAC GDE. (b) Contact angles of UFe_3_O_4_@HPAC GDE prepared by curing the catalyst with polytetrafluoroethylene (PTFE) emulsion and Nafion solution, respectively.


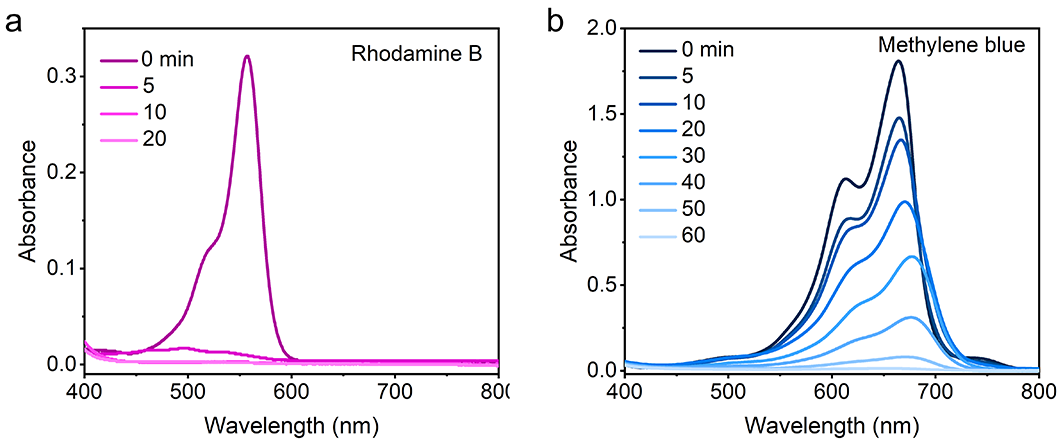


Figure S17. UV–visible absorption spectra of (a) RhB, and (b) MB.


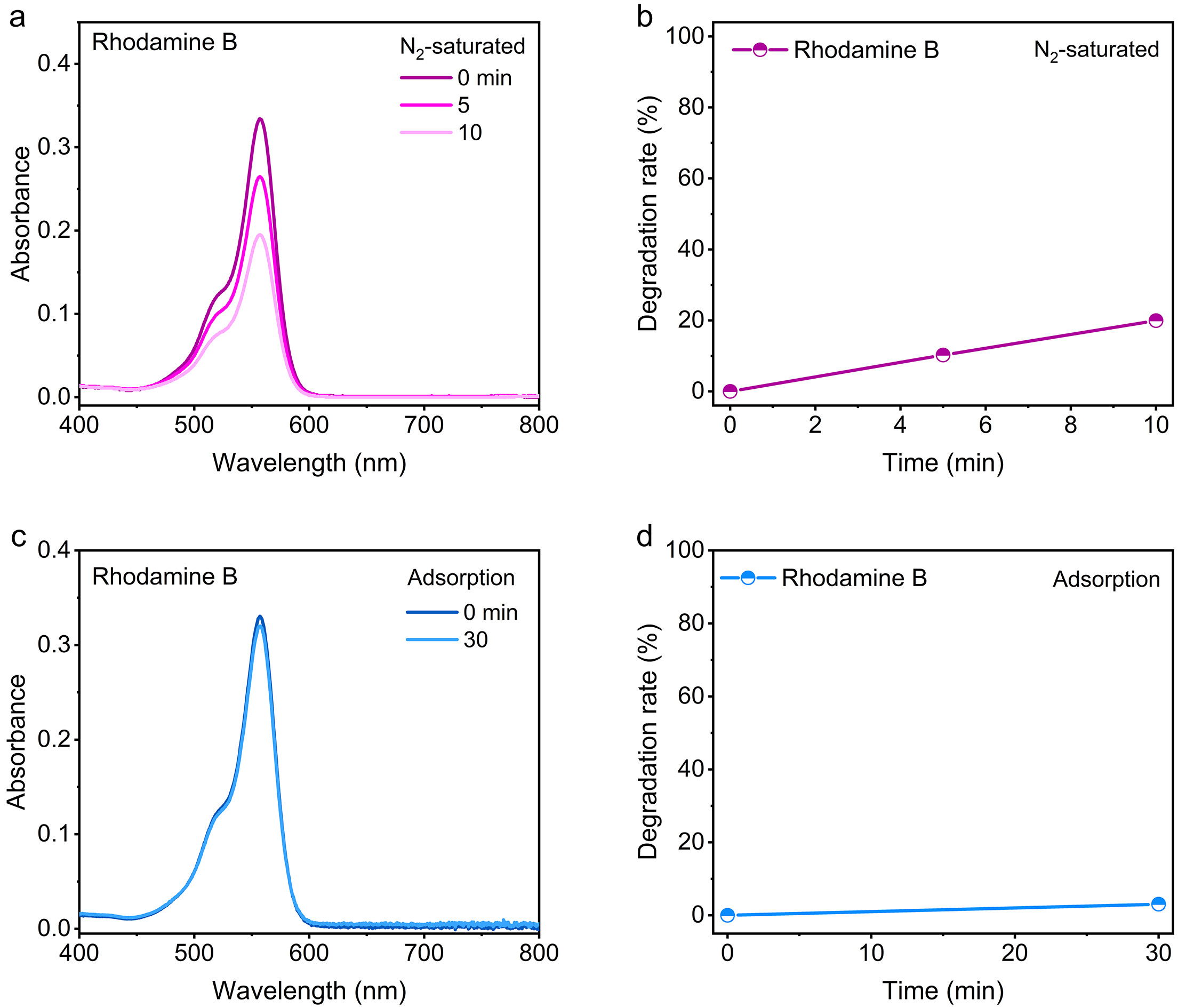


Figure S18. (a) UV–visible absorption spectra and (b) degradation efficiency of a 500 mg L^–1^ RhB solution in N_2_-saturated 0.5 M Na_2_SO_4_ over time in electro-Fenton processes. (c) UV–visible absorption spectra and (d) adsorption capacity of UFe_3_O_4_@HPAC GDE for a 500 mg L^–1^ RhB solution within 30 min.


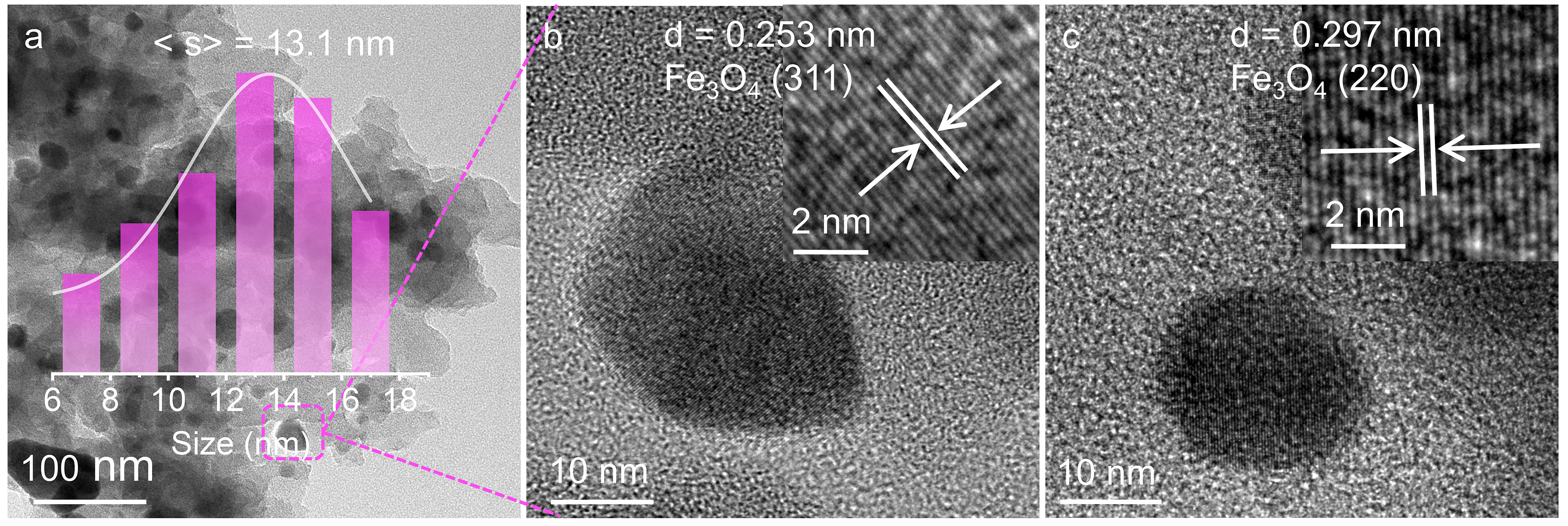


Figure S19. Structural characterization of the catalyst after 18 consecutive cycles of electro-Fenton degradation: (a) TEM image and Fe_3_O_4_ particle size distribution, (b) (c) HRTEM images.


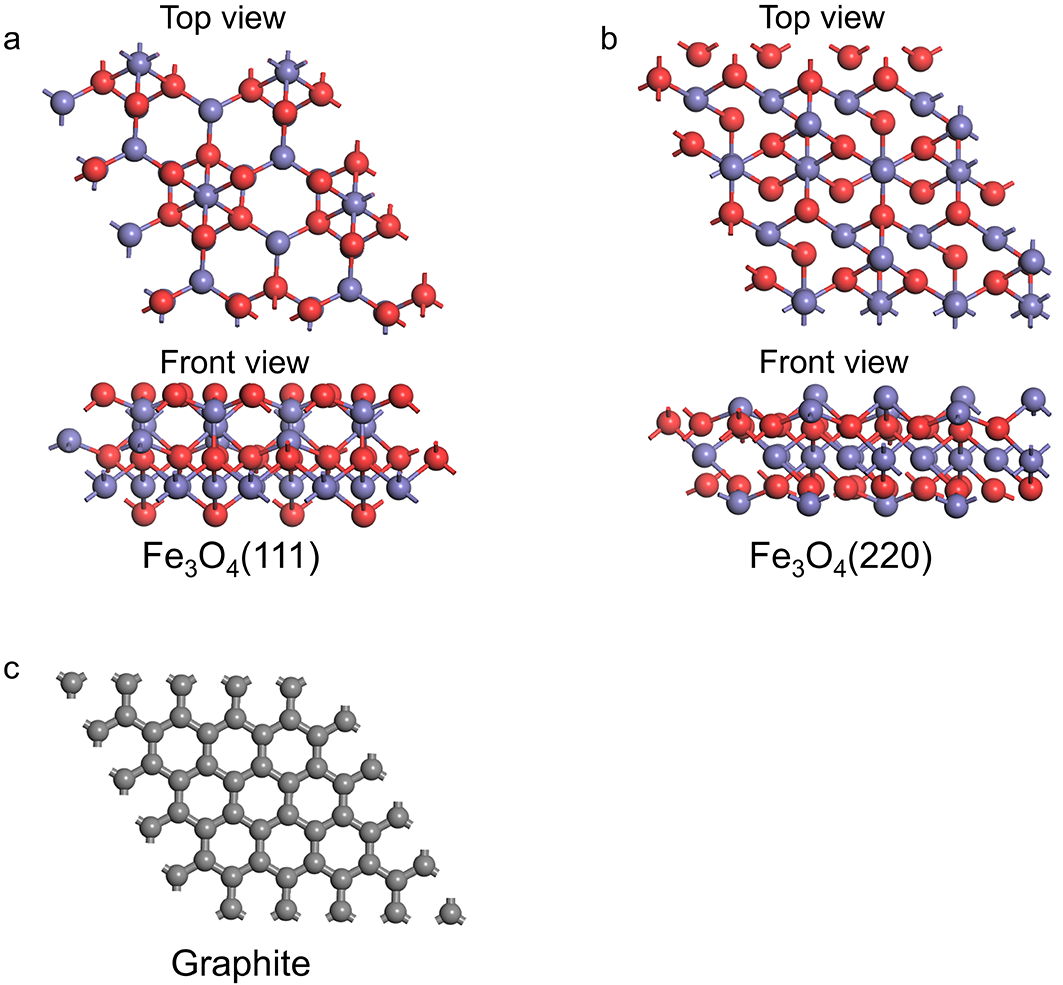


Figure S20. Models for Fe_3_O_4_(111), Fe_3_O_4_(220), and Graphite. The gray, red, and purple balls represent C, O, and Fe atoms, respectively.


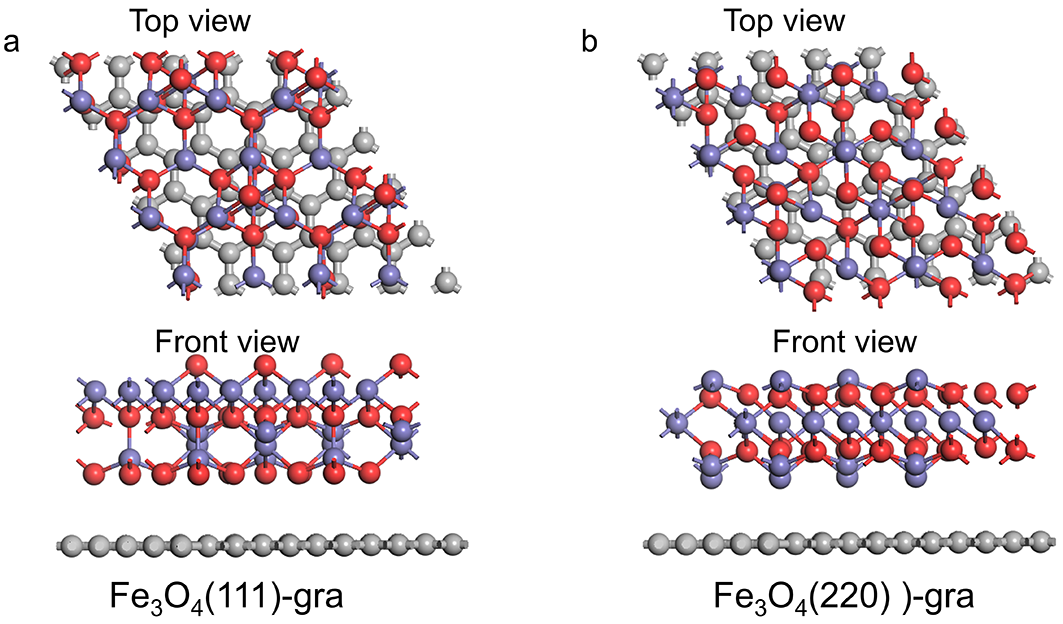


Figure S21. Models for Fe_3_O_4_(111)-gra, and Fe_3_O_4_(220)-gra. The gray, red, and purple balls represent C, O, and Fe atoms, respectively.


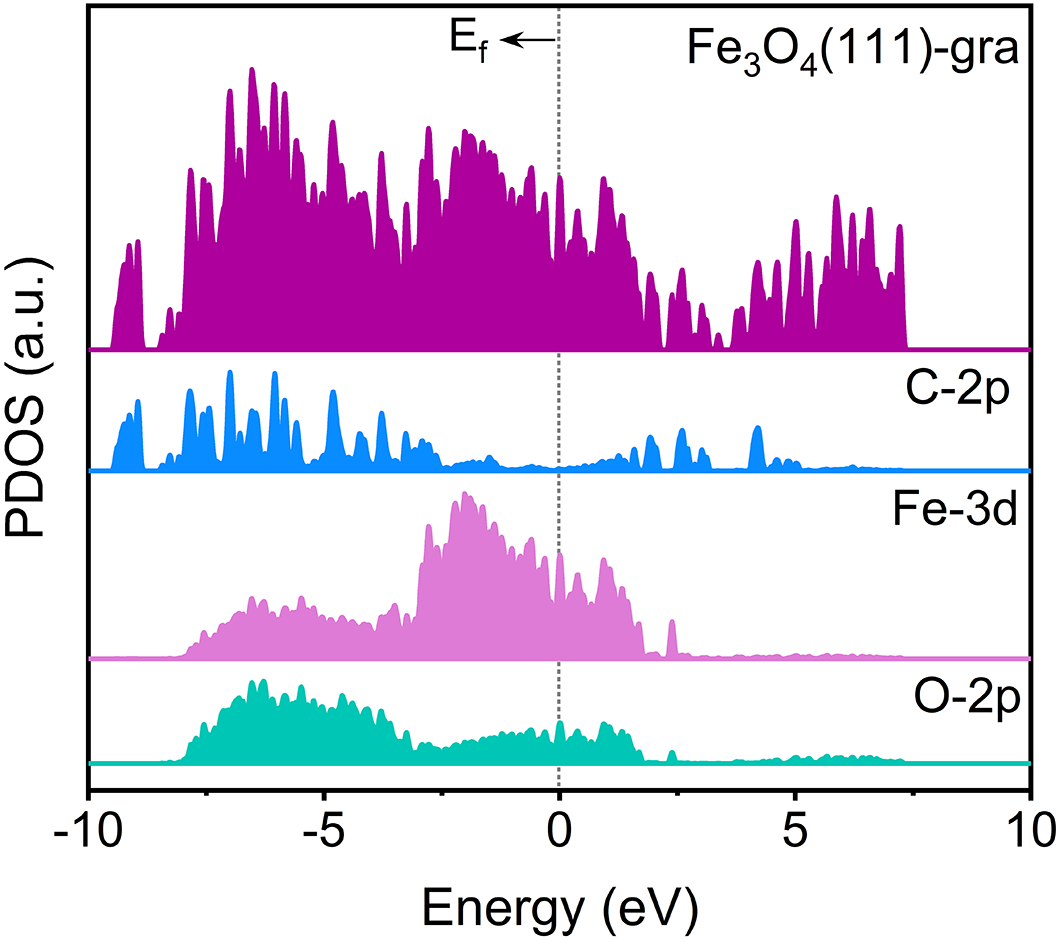


Figure S22. Total DOS of unit cells of Fe_3_O_4_(111)-gra.


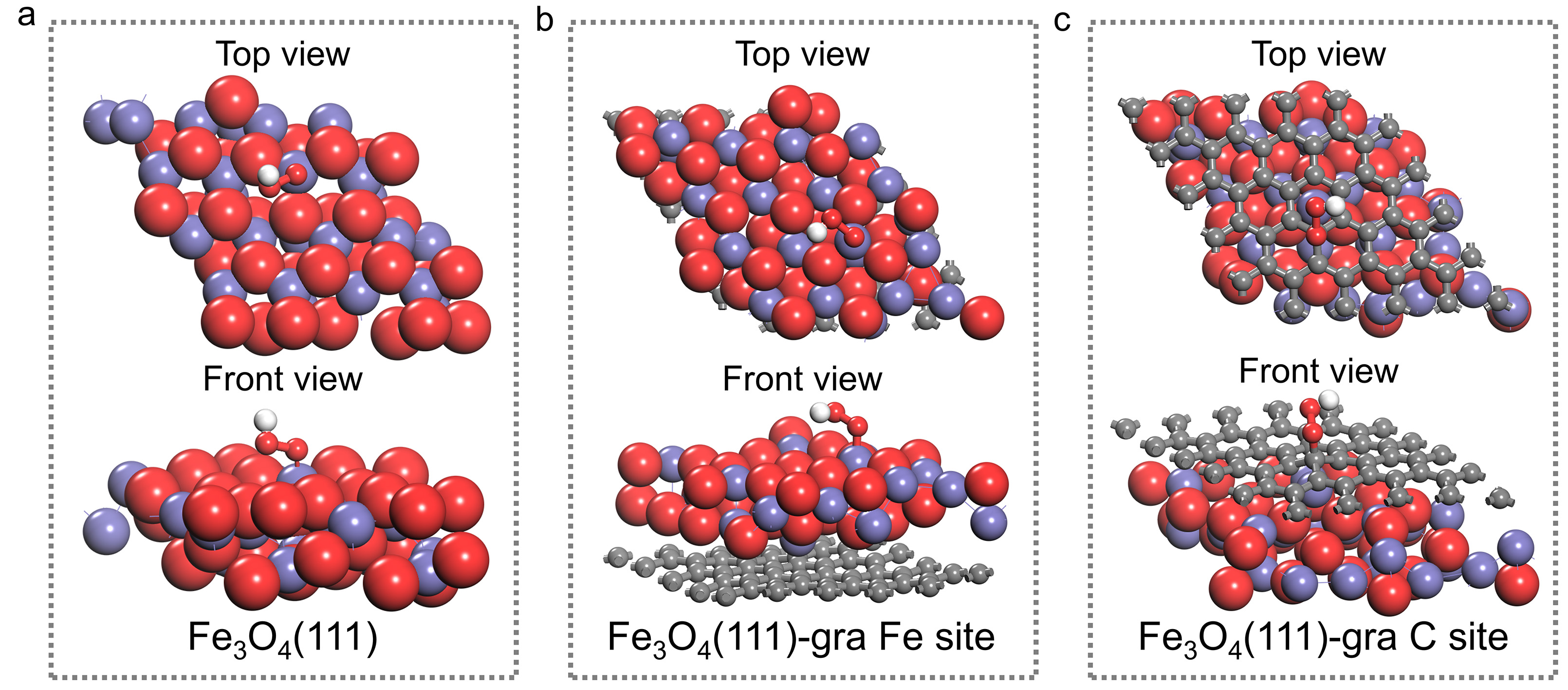


Figure S23. Optimized *OOH adsorption models.


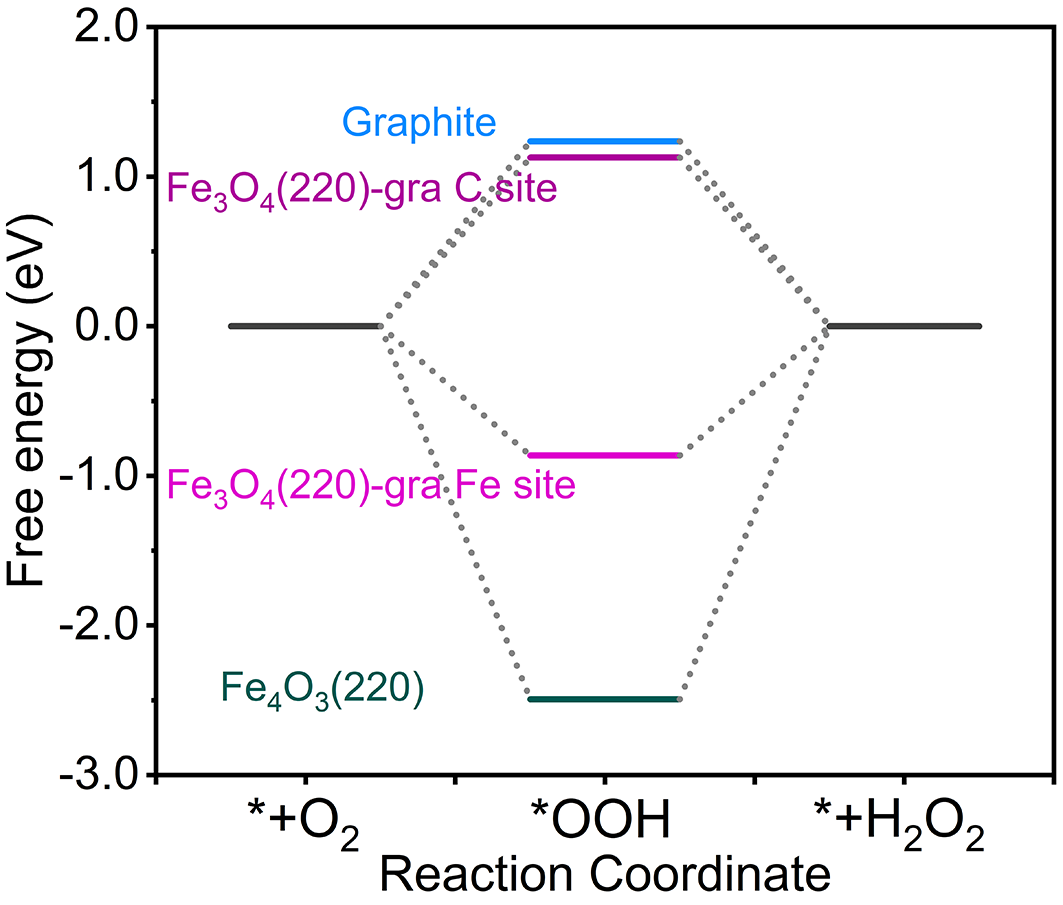


Figure S24. Free energy profile of 2e^–^ ORR pathways at the equilibrium potentials of 0.7 V vs. RHE.

Table S1. The mass loading of Fe by inductively coupled plasma mass spectrometer (ICP-MS) and Energy Dispersive X-ray (EDX).

| Catalysts | by ICP-MS (wt%) | by EDX (at%) |
| --- | --- | --- |
| HPAC | 0.11 | 0.02 |

Table S2. Summary of binding energy and relative element contents from the deconvoluted XPS spectra of the samples.

| Catalysts | Binding energy (eV) | | | XPS (at%)^a^ |
| --- | --- | --- | --- | --- |
|  | C | O | Fe | Fe |
| HPAC | 284.80 | 532.65 | --- | 0 |
| UFe_3_O_4_@HPAC | 284.77 | 530.44 | 710.40 | 0.78 |
| Fe_3_O_4_ | ---- | 530.19 | 710.76 | ---- |

(a) Atomic percentages (at%) obtained from XPS.

Table S3. Comparison of the H_2_O_2_ selectivity and onset potential at a disk current 0.1 mA cm^–2^ in alkaline media for UFe_3_O_4_@HPAC versus previously reported carbon catalysts with nitrogen doping, oxygen doping, or nitrogen-oxygen co-doping.

| Catalysts | Electrolyte | Onset potential  (V vs. RHE) | | H_2_O_2_ Selectivity (%) | References |
| --- | --- | --- | --- | --- | --- |
| UFe_3_O_4_@HPAC | 0.1 M KOH | 0.71 | 90–96 | | This work |
| ZnO | 0.1 M KOH | 0.70 | ~90 | | J. Mater. Chem. A 2024 [5] |
| {001}-Fe_2_O_3_-x | 0.1 M KOH | 0.73 | >95 | | Adv. Funct. Mater. 2020 [6] |
| NiO_x_-C | 0.1 M KOH | 0.76 | ~91 | | ACS Catal. 2022 [7] |
| Ni_4_-B1@BNC | 0.1 M KOH | 0.63 | ~86 | | Small 2022 [8] |
| NiZnMOF | 0.1 M KOH | 0.73 | ~90 | | Small 2022 [9] |
| NiFe-MOFs | 0.1 M KOH | 0.60 | ~90 | | Appl. Catal., B 2024 [10] |
| Cu-TiO_2_ | 0.1 M KOH | 0.79 | 87–91.2 | | Nano Res. 2022 [11] |
| a-TiO_2-x_/TiC | 0.1 M KOH | 0.75 | 90–93 | | ACS Appl. Mater. Interfaces 2021 [12] |
| CuO_x_-G-30 | 1 M KOH | A/N | ~80 | | Chem. Commun. 2021 [13] |
| Fe_3_O_4_/graphene | 1 M KOH | A/N | 68 | | Electrochim. Acta 2015 [14] |
| Fe_3_O_4_/Printex | 1 M KOH | A/N | 62 | | Electrochim. Acta 2015 [14] |
| Nb_2_O_5_-rGO | 0.1 M NaOH | 0.68 | 74.9 | | J. Catal. 2015 [15] |

Table S4. Fe content in 0.1 M KOH, measured by inductively coupled plasma mass spectrometry (ICP-MS), after 5,000 CV cycles in the accelerated degradation test for UFe_3_O_4_@HPAC.

| Sample | by ICP-MS (mg L^–1^) |
| --- | --- |
| 0.1 M KOH electrolyte | 0.075 |

**References**

[1] Y. Shi, C. Wang, L. Zhang, S. Sun, *ACS Appl. Nano Mater.* **2024**, *7*, 13328.

[2] a) G. Kresse, J. Hafner, *Phys. Rev. B.* **1994**, *49*, 14251; b) G. Kresse, J. Furthmu¨ller, *Phys. Rev. B.* **1996**, *54*, 11169; c) G. Kresse, J. Furthmuller, *Comput. Mater. Sci.* **1996**, *6*, 15.

[3] a) J. P. Perdew, K. Burke, M. Ernzerhof,  *Phys. Rev. Lett.* **1996**, *77*, 3865; b) M. Ernzerhof, J. P. Perdew, *J. Chem. Phys.* **1998**, *109*, 3313.

[4] J. R. J. K. Nørskov, A. Logadottir, and L. Lindqvist, *J. Phys. Chem. B.* **2004**, 17886.

[5] Y. Zhang, H. Jiang, C. Zhang, Y. Feng, H. Feng, S. Zhu, J. Hu, *J. Mater. Chem. A.* **2024**, *12*, 6123.

[6] R. Gao, L. Pan, Z. Li, C. Shi, Y. Yao, X. Zhang, J. J. Zou, *Adv. Funct. Mater.* **2020**, *30*, 1910539.

[7] Z. Wu, T. Wang, J.-J. Zou, Y. Li, C. Zhang, *ACS Catal.* **2022**, *12*, 5911.

[8] H. Fu, N. Zhang, F. Lai, L. Zhang, Z. Wu, H. Li, H. Zhu, T. Liu, *Small.* **2022**, *18*, 2203510.

[9] M. Liu, H. Su, W. Cheng, F. Yu, Y. Li, W. Zhou, H. Zhang, X. Sun, X. Zhang, S. Wei, Q. Liu, *Small.* **2022**, *18*, 2202248.

[10] H.-T. Tang, Z.-J. Tang, R.-L. Li, L. Tian, R. Wang, S. Pu, Z.-Q. Liu, *Appl. Catal., B.* **2024**, *358*, 124436.

[11] Z. Deng, L. Li, Y. Ren, C. Ma, J. Liang, K. Dong, Q. Liu, Y. Luo, T. Li, B. Tang, Y. Liu, S. Gao, A. M. Asiri, S. Yan, X. Sun,  *Nano Res.* **2022**, *15*, 3880.

[12] Z. Xu, J. Liang, Y. Wang, K. Dong, X. Shi, Q. Liu, Y. Luo, T. Li, Y. Jia, A. M. Asiri, Z. Feng, Y. Wang, D. Ma, X. Sun, *CS Appl. Mater. Interfaces.* **2021**, *13*, 33182.

[13] H. Xiao, B. Li, M. Zhao, Y. Li, T. Hu, J. Jia, H. Wu, *Chem. Commun.* **2021**, *57*, 4118.

[14] W. R. P. Barros, Q. Wei, G. Zhang, S. Sun, M. R. V. Lanza, A. C. Tavares, *Electrochim. Acta.* **2015**, *162*, 263.

[15] J. F. Carneiro, M. J. Paulo, M. Siaj, A. C. Tavares, M. R. V. Lanza, *J. Catal.* **2015**, *332*, 51.
